# Epidemiology of capybara-associated Brazilian spotted fever

**DOI:** 10.1371/journal.pntd.0007734

**Published:** 2019-09-06

**Authors:** Hermes R. Luz, Francisco B. Costa, Hector R. Benatti, Vanessa N. Ramos, Maria Carolina de A. Serpa, Thiago F. Martins, Igor C. L. Acosta, Diego G. Ramirez, Sebastián Muñoz-Leal, Alejandro Ramirez-Hernandez, Lina C. Binder, Marcio Port Carvalho, Vlamir Rocha, Thiago C. Dias, Camila L. Simeoni, José Brites-Neto, Jardel Brasil, Ana Maria Nievas, Patricia Ferreira Monticelli, Maria Estela G. Moro, Beatriz Lopes, Daniel M. Aguiar, Richard C. Pacheco, Celso Eduardo Souza, Ubiratan Piovezan, Raquel Juliano, Katia Maria P. M. B. Ferraz, Matias P. J. Szabó, Marcelo B. Labruna

**Affiliations:** 1 Departamento de Medicina Veterinária Preventiva e Saúde Animal, Faculdade de Medicina Veterinária e Zootecnia, Universidade de São Paulo, São Paulo, SP, Brazil; 2 Departamento de Patologia, Programa de Pós Graduação em Biotecnologia do Renorbio, Ponto Focal Maranhão, Universidade Federal do Maranhão, São Luís, MA, Brazil; 3 Departamento de Patologia, Faculdade de Medicina Veterinária, Universidade Estadual do Maranhão, São Luís, MA, Brazil; 4 Pós-Doutorado em Ciências Veterinárias, Faculdade de Medicina Veterinária, Universidade Federal de Uberlândia, Uberlândia, MG, Brazil; 5 Departamento de Parasitologia Animal, Instituto de Medicina Veterinária, Universidade Federal Rural do Rio de Janeiro, Seropédica, RJ, Brazil; 6 Instituto Florestal, São Paulo, SP, Brazil; 7 Centro de Ciências Agrárias, Universidade Federal de São Carlos, Araras, SP, Brazil; 8 Programa de Pós-graduação em Ecologia e Recursos Naturais, Centro de Ciências Biológicas e da Saúde, Universidade Federal de São Carlos, São Carlos, SP, Brazil; 9 Departamento de Vigilância Epidemiológica, Secretaria Municipal de Saúde, Americana, SP, Brazil; 10 Departamento de Psicologia, Faculdade de Filosofia, Ciências e Letras de Ribeirão Preto, Universidade de São Paulo, Ribeirão Preto, SP, Brazil; 11 Departamento de Zootecnia, Faculdade de Zootecnia e Engenharia de Alimentos, Universidade de São Paulo, Pirassununga, SP, Brazil; 12 Departamento de Ciências Florestais, Escola Superior de Agricultura Luiz de Queiroz, Universidade de São Paulo, Piracicaba, SP, Brazil; 13 Programa de Pós-graduação em Ciências Veterinárias, Faculdade de Medicina Veterinária, Universidade Federal de Mato Grosso, Cuiabá, MT, Brazil; 14 Laboratório de Carrapatos, Superintendência de Controle de Endemias, Mogi Guaçu, SP, Brazil; 15 Embrapa Pantanal, Corumbá, MS, Brazil; 16 Embrapa Tabuleiros Costeiros, Aracaju, SE, Brazil; 17 Laboratório de Ixodologia, Faculdade de Medicina Veterinária, Universidade Federal de Uberlândia, Uberlândia, MG, Brazil; Baylor College of Medicine, UNITED STATES

## Abstract

**Background:**

Brazilian spotted fever (BSF), caused by the bacterium *Rickettsia rickettsii*, has been associated with the transmission by the tick *Amblyomma sculptum*, and one of its main hosts, the capybara (*Hydrochoerus hydrochaeris*).

**Methods:**

During 2015–2019, we captured capybaras and ticks in seven highly anthropic areas of São Paulo state (three endemic and four nonendemic for BSF) and in two natural areas of the Pantanal biome, all with established populations of capybaras.

**Results:**

The BSF-endemic areas were characterized by much higher tick burdens on both capybaras and in the environment, when compared to the BSF-nonendemic areas. Only two tick species (*A*. *sculptum* and *Amblyomma dubitatum*) were found in the anthropic areas; however, with a great predominance of *A*. *sculptum* (≈90% of all ticks) in the endemic areas, in contrast to a slight predominance of *A*. *dubitatum* (≈60%) in the nonendemic areas. Tick species richness was higher in the natural areas, where six species were found, albeit with a predominance of *A*. *sculptum* (≈95% of all ticks) and environmental tick burdens much lower than in the anthropic areas. The BSF-endemic areas were characterized by overgrowth populations of *A*. *sculptum* that were sustained chiefly by capybaras, and decreased populations of *A*. *dubitatum*. In contrast, the BSF-nonendemic areas with landscape similar to the endemic areas differed by having lower tick burdens and a slight predominance of *A*. *dubitatum* over *A*.*sculptum*, both sustained chiefly by capybaras. While multiple medium- to large-sized mammals have been incriminated as important hosts for *A*. *sculptum* in the natural areas, the capybara was the only important host for this tick in the anthropic areas.

**Conclusions:**

The uneven distribution of *R*. *rickettsii* infection among *A*. *sculptum* populations in highly anthropic areas of São Paulo state could be related to the tick population size and its proportion to sympatric *A*. *dubitatum* populations.

## Author summary

Brazilian spotted fever (BSF), caused by the bacterium *Rickettsia rickettsii*, is the deadliest tick-borne disease of the New World. In southeastern Brazil, where 489 patients succumbed to the disease from 2001 to 2018, *R*. *rickettsii* is transmitted to humans mainly by the tick *Amblyomma sculptum*, which uses the capybara (*Hydrochoerus hydrochaeris*) as its main host. During 2015–2019, we captured capybaras and ticks in seven highly anthropic areas of São Paulo state (three endemic and four nonendemic for BSF) and in two natural areas of the Pantanal biome. The BSF-endemic areas were characterized by much higher tick burdens on both capybaras and in the environment, with a predominance of *Amblyomma sculptum*. In the BSF-nonendemic areas, another tick species, *Amblyomma dubitatum*, outnumbered *A*. *sculptum*. In the natural areas, six tick species were found; however, with much lower numbers than in the anthropic areas. The BSF-endemic areas were characterized by overgrowth populations of *A*. *sculptum* that were sustained chiefly by capybaras, and decreased populations of *A*. *dubitatum*. Results of this study support the idea that any intervention resulting in a drastic reduction of the *A*. *sculptum* population shall eliminate the *R*. *rickettsii* infection from the tick population, and consequently, prevent new BSF cases.

## Introduction

Brazilian spotted fever (BSF), caused by the bacterium *Rickettsia rickettsii*, is the deadliest tick-borne disease of the New World. The disease is endemic in many parts of southeastern Brazil, especially in the state of São Paulo, where 978 laboratory-confirmed cases were recorded from 2001 to 2018, of which 489 (50%) had a fatal outcome (official data from the São Paulo State Health Secretary). In North America, where the *R*. *rickettsii-*caused disease is known as Rocky Mountain spotted fever (RMSF), multiple strains of *R*. *rickettsii* (including less virulent ones) are known to occur. In contrast, a highly virulent strain prevails in Central and South America, which has been linked to the higher fatality rates of BSF, when compared to RMSF [1]. In addition, the greatest fatality of BSF is also evidenced by its neglected status in Brazil, such as the unavailability in the country of parenteral doxycycline, considered the first-choice medication to treat severe BSF or RMSF presenting vomiting or altered mental status [2, 3].

During this century, several studies have elucidated key factors in the epidemiology of BSF in southeastern Brazil, where *R*. *rickettsii* is transmitted to humans mainly by the tick *Amblyomma sculptum*. Besides being a competent vector, *A*. *sculptum* larvae, nymphs and adults are partially refractory to *R*. *rickettsii* infection, and less than half of the infected females transmit *R*. *rickettsii* to their offspring (transovarial transmission) [4–6]. This fact, associated to the higher mortality and lower reproductive performance of infected ticks, when compared to uninfected mates (5, 6), causes infection of *A*. *sculptum* by *R*. *rickettsii* in BSF-endemic areas to be very low, usually <1% [7–10]. Within this scenario, mathematical models have indicated that an *A*. *sculptum* population is not able to sustain a *R*. *rickettsii* infection for successive tick generations without the creation of new cohorts of infected ticks via horizontal transmission on vertebrate rickettsemic hosts (amplifying hosts) [11, 12]. In this case, the capybara (*Hydrochoerus hydrochaeris*), the largest living rodent in the world, has been pointed out as the major amplifying host of *R*. *rickettsii* for *A*. *sculptum* in most of the BSF-endemic areas of southeastern Brazil [11, 13, 14]. However, it is important to note that the tick *Amblyomma dubitatum* has also been frequently found infesting capybaras in southeastern Brazil, albeit with no direct role on BSF-epidemiology [7–10, 14, 15].

During the last four decades, the state of São Paulo has undergone extensive anthropogenic modifications in its landscape due to a rapid expansion of agricultural crops (especially sugar cane), deforestation, and creation of artificial water bodies [16, 17]. Such modifications have favored capybara reproduction primarily by higher food availability by agriculture (e.g., sugar cane, corn fields) and because of the local extinction of natural predators (e.g., the jaguar *Panthera onca*), in human-modified landscapes, leading to an increment on the extension and density of its populations [17–19]. Because capybara is considered to be the main host for *A*. *sculptum* in such landscapes [10, 15, 20], and at the same time an efficient *R*. *rickettsii* amplifying host [13], the increase of BSF incidence in the state of São Paulo during the last three decades has been associated to the afore mentioned anthropogenic modifications [11, 14, 21].

While the expansion of capybaras and their ticks have been well recognized in the state of São Paulo during the last decades, many of these human-modified landscapes have remained free of *R*. *rickettsii* circulation, despite of the established presence of capybaras and *A*. *sculptum* [22–24]. Since the reasons determining the establishment of *R*. *rickettsii* in a capybara-sustained *A*. *sculptum* population are not well understood, the present study aimed to characterize and to quantify in time and space the tick fauna in capybaras and in the habitats where these rodents occur among different human-modified landscapes in the state of São Paulo, either endemic or nonendemic for BSF. Differences in the tick fauna composition could be one of the possible multiple reasons driving the uneven distribution of *R*. *rickettsii* among different *A*. *sculptum* populations. In order to confirm the endemic or nonendemic status of each area, we determined the serological profile of the capybaras against a battery of rickettsial antigens, including *R*. *rickettsii*. For comparison purposes, we performed the same capybara and tick evaluations in pristine areas of the Pantanal biome of Brazil, where capybaras live in natural habitats in which landscape has suffered only minimal anthropogenic alterations and from where BSF has never been reported. Our results might provide some clues for a better understanding on the main epidemiological characteristics of the BSF-endemic areas associated to capybaras.

## Methods

### Ethical statements

This study has been approved by the Institutional Animal Care and Use Committee (IACUC) of the Faculty of Veterinary Medicine of the University of São Paulo (approval number 5948070314), in accordance with the regulations/guidelines of the Brazilian National Council of Animal Experimentation (CONCEA). Field capture of capybaras and collections of ticks were authorized by the Brazilian Ministry of the Environment (permit SISBIO Nos. 43259–6) and by the São Paulo Forestry Institute (Cotec permit 260108–000.409/2015).

### Study areas

**Fig 1:**
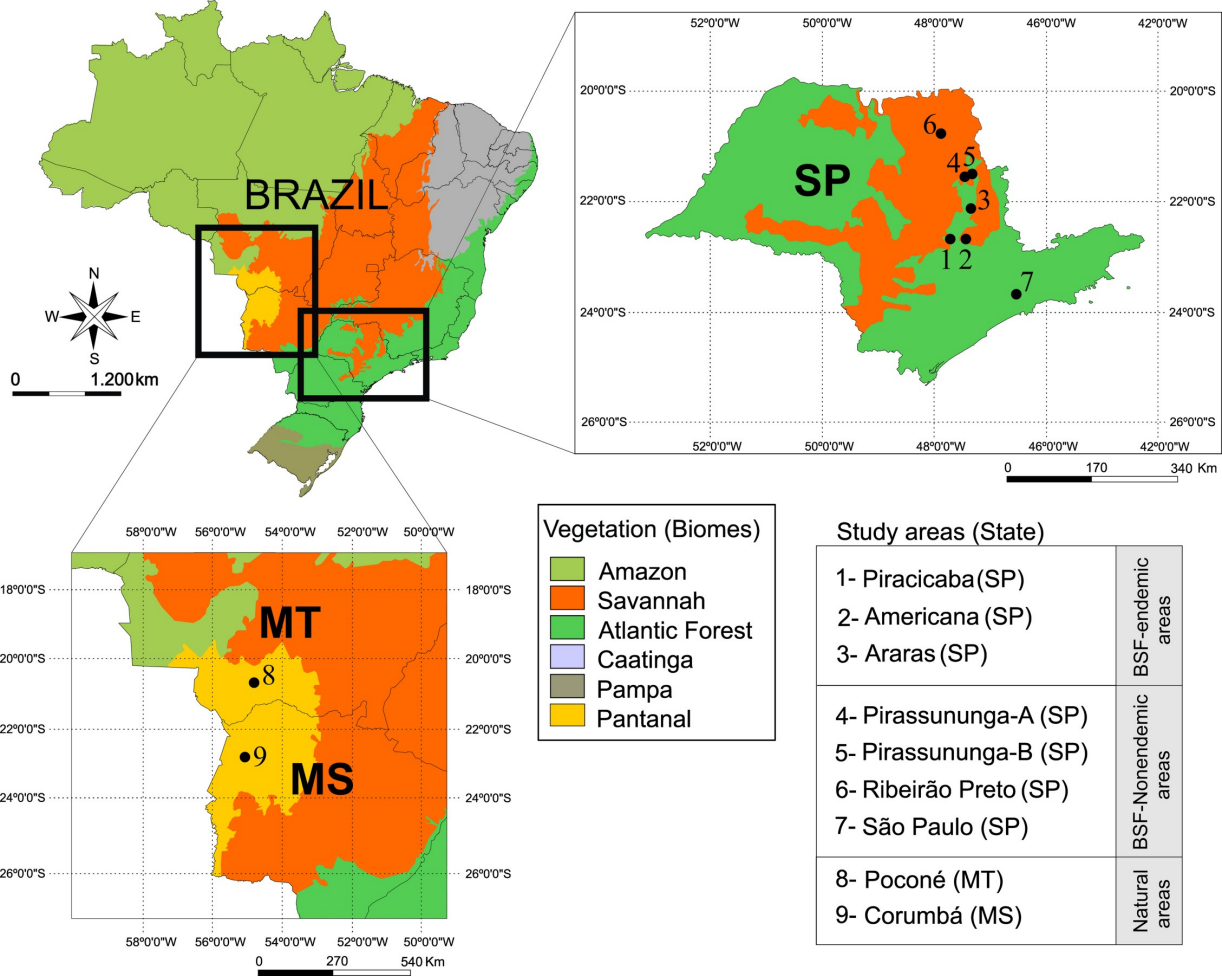
Areas where capybaras and ticks were sampled in the state of São Paulo (SP) (highly anthropic, low diversity areas), and in the states of Mato Grosso (MT) and Mato Grosso do Sul (MS) (low anthropic, high diversity areas). Map source was obtained from the “Instituto Brasileiro de Geografia e Estatística”(IBGE) website (www.ibge.gov.br) and the final figure was constructed with the use of CorelDraw Graphics Suite 2017.

All study areas were inhabited by capybaras, and were classified into the following three epidemiological categories: (i) BSF-endemic areas–highly anthropic areas (human-modified landscape) in the state of São Paulo, where human cases of BSF have been recently confirmed and the transmissions have been epidemiologically associated with *A*. *sculptum*. Three BSF-endemic areas were sampled: Area 1 in the municipality of Piracicaba, Area 2 in the municipality of Americana, Area 3 in the municipality of Araras, all located in transition areas of the biomes Savannah and Atlantic Forest; (ii) BSF-nonendemic areas–highly anthropic areas (human-modified landscape) in the state of São Paulo, however, with no history of BSF. Four BSF-nonendemic areas were sampled: Areas 4 and 5 in the municipality of Pirassununga, located in a transition area of the biomes Savannah and Atlantic Forest, Area 6 in the municipality of Ribeirão Preto, located in the Savannah biome, and Area 7 in São Paulo municipality, in the Atlantic Forest biome; and (iii) natural areas–low anthropic areas (natural landscape) in the Pantanal biome, with no history of BSF. Two natural areas were sampled: Area 8 in Poconé municipality, state of Mato Grosso, and Area 9 in Corumbá municipality, state of Mato Grosso do Sul. We sampled capybaras and ticks in 3 BSF-endemic areas, 4 BSF-nonendemic areas, and 2 natural areas (S1 Table, Fig 1 and S1 Text).

### Capybara sampling

During 2015–2018, we performed capture of capybaras in all study areas by using 16 to 20 m^2^–corrals baited with sugar cane and green corn. Once closed in the corral, every animal was physically restrained by a net catcher and anaesthetized with an intramuscular injection of a combination of ketamine (10 mg/kg) and xylazine (0.2 mg/kg). Under anesthesia, animals were weighed in an electronic balance (Pesola model PCS0300, Hatton Rock, UK) and identified with a subcutaneous microchip (Alflex model P/N 860005–001, Capalaba, Australia). In Mato Grosso do Sul, corrals were not effective, thus capybaras were captured by anesthetic darting via a CO_2_-injection rifle (Dan-Inject model JM Standard, Denmark) by injecting the same chemicals above. Capybaras were sexed and aged as follows: young (<10 Kg), juvenile (10–35 Kg), and adult (>35 Kg), following Vargas et al. [25]. From each capybara, we collected blood samples through the femoral artery or cranial vena cava, and the serum was separated by centrifugation and stored frozen at -20°C until serological analysis (described below). Because many capybaras were heavily infested by ticks, we standardized a 3-min random collection of ticks from the entire body of every capybara. During the 3-min period, any tick on sight was collected, regardless of the size or part of capybara body. These ticks were brought to the laboratory, where they were identified to species following current literature [20, 26, 27]. After recovering from anesthesia, capybaras were released at the same capture site.

### Serological analysis

Capybara sera were tested by immunofluorescence assay (IFA) as described elsewhere [22] using rickettsial crude antigens derived from Vero cells (provided by the Instituto Adolfo Lutz, São Paulo, Brazil) infected with each of the following five *Rickettsia* species known to infect ticks in Brazil: *R*. *rickettsii* strain Taiaçu [28], *Rickettsia parkeri* strain At24 [29], *Rickettsia amblyommatis* strain Ac37 [30], *Rickettsia rhipicephali* strain HJ5 [31], and *Rickettsia bellii* strain Mogi [28]. In addition, a sixth rickettsial antigen consisted of C6/36 cells (provided by the Instituto Adolfo Lutz, São Paulo, Brazil) infected with *Rickettsia felis* strain Pedreira, was also implemented [32]. In each slide, a serum previously shown to be non-reactive (negative control) and a known reactive serum (positive control) from a previous study [13] were included. Slides were incubated with fluorescein isothiocyanate-labeled sheep anti-capybara IgG (produced by the Centro de Controle de Zoonoses, São Paulo City). For each sample, the endpoint titer reacting with each of the six *Rickettsia* antigens was determined. Sera showing an antibody titer to a *Rickettsia* species at least fourfold higher than the titers observed for the other *Rickettsia* species were supposed to be homologous to the first *Rickettsia* species or to a very closely related genotype, as previously determined for several animal species [33–35], including capybaras [22].

### Collection of host-questing ticks

Host questing ticks were collected in each of the nine study areas (Fig 1, S1 Table) during four consecutive years. Our schedule for collection of free-living ticks was based on the seasonal dynamics of *A*. *sculptum*, which is known to complete one generation per year, with larvae peaking during autumn, nymphs during spring, and adults during summer [36–38]. Therefore, between May 2015 and January 2019, ticks were collected in each area during the larval peak (May-June), nymphal peak (August-September) and adult peak (January-February) of every year. In each area at each time point, a 1 m^2^ white flannel was dragged over 800 m of animal trails. With this procedure, every dragging event on a given area represented the number of ticks for an 800 m^2^-sampled area. Collected nymphs and adults were immediately put in plastic vials containing 70% ethanol, except for a few adult ticks that were placed in dry plastic vials and taken alive to the laboratory, where they were kept frozen at -80°C until molecular analysis for *Rickettsia* (described below). Every time a larval cluster was captured by dragging, the cluster was immediately picked up with a 5 cm-large transparent plastic adhesive tape, which was then stuck on a white paper that was put within a sealed plastic bag and taken to the laboratory. Adult and nymphal ticks were counted individually and identified to species according to [20, 26, 27]. Larvae were counted as number of clusters, since it was assumed that each larval cluster represented the offspring of one engorged female [38, 39]. Larval taxonomic identification consisted of comparing side-by-side individuals of a larval cluster with laboratory-reared larvae of *A*. *sculptum* and *A*. *dubitatum*, following established criteria [40, 41].

Host-questing ticks were also collected by dry ice traps following Szabó et al. [39]; however, this method was used only at one time point (August 2015) in each area, and had to be discontinued due to logistic difficulties. In each area, 20 to 40 dry ice traps were set at 10 m intervals along the same trails that were sampled by dragging. Collected ticks were immediately placed in 70% ethanol, and taken to the laboratory for taxonomic identification as described above.

### Rickettsial detection in ticks

Frozen unfed adult ticks, previously collected by dragging in each area, were thawed and individually submitted to DNA extraction by the guanidine isothiocyanate-phenol technique [42]. Extracted DNA samples were firstly tested by a conventional PCR protocol targeting the tick mitochondrial 16S rRNA gene, as previously described [43], in order to certify successful DNA extraction. Then, viable DNA samples (those positive by the tick 16S rRNA PCR assay) were tested by a Taqman real-time PCR assay targeting the rickettsial *gltA* gene, as described [5]. The sensitivity of this PCR assay was determined to be 1 DNA copy of *R*. *rickettsii* [44]. Positive samples by this Taqman real-time PCR were tested by two protocols of conventional PCR, one targeting a 401-bp fragment of the rickettsial *gltA* gene [44], and one heminested PCR assay targeting the *ompA* gene; this latter protocol consisted of a first reaction targeting a 631-bp fragment, and a second targeting a 532-bp fragment, as described [45]. PCR products were DNA-sequenced and the resultant sequences were submitted to BLASTn analyses (www.ncbi.nlm.nih.gov/blast) in order to confirm the identity of the *Rickettsia* species.

### Data analyses

The proportions of seroreactive capybaras for *R*. *rickettsii* were compared between the nine sampled areas by the Chi-square test. Endpoint titers for the six *Rickettsia* species were compared between BSF-endemic and BSF-nonendemic areas by the Mann-Whitney test. For the ticks collected on capybaras, we calculated the prevalence (No. infested hosts / No. examined capybaras x 100), and the mean abundance of tick infestation (total number of collected ticks / number of examined capybaras) according to [46] in each of the 9 study areas.

Density of host-questing ticks was calculated for the total dragged area (TDA). For this purpose, TDA = number of dragging events performed in one area during the four years of study x 800m^2^ (considering that each dragging event encompassed an 800m^2^ area in each of the study areas). Then, the tick density (TD), represented by number of host-questing ticks per 1,000 m^2^, was calculated by: TD = total number of collected ticks / TDA x 1,000 m^2^. These calculations were applied to the two most abundant tick species, *A*. *sculptum* and *A*. *dubitatum*. TD was calculated separately for larvae, nymphs, and adult ticks for the whole study period, as well as for the period of larval peak (all collections during autumn), nymphal peak (all collections during winter), and adult peak (all collections during summer).

For statistical analyses, we pooled the tick data for each of the three epidemiological categories: (i) BSF-endemic areas, (ii) BSF-nonendemic areas, and (iii) natural areas. The Chi-square test was used to compare the differences between the proportions of ticks on capybaras or host-questing ticks between the three epidemiological categories (BSF-endemic, BSF-nonendemic, natural areas). The Lilliefors test implemented in the PAST 3.19 program was used to analyze the normality of the data in order to choose the appropriate statistical test for each situation. Values of mean abundance of tick infestation were analyzed using the Kruskal-Wallis non-parametric test. For all tests, the level of significance was 5%. Analyses were performed by using PAST Version 3.19 and BioEstat 5.0.

## Results

### Captured capybaras

A total of 347 capybaras were captured during the 2015–2018 period. The number of captured capybaras per each of the nine areas varied from 14 to 73 (mean: 38.6). Since we sampled during four consecutive years, some individuals were captured twice among different years; recaptured capybaras represented 0 to 50% of the total number of captures in each area (Table 1). Removing recaptured animals, the total number of different individuals sampled in this study was 287; however, we considered all recaptures as different units for our analyses of serology and tick infestations (described below), since recaptures occurred in years different from the first capture. The 347 captured capybaras were represented by 94 (27%) males and 253 (73%) females. They were aged as 27 (7.8%) young, 70 (20.2%) juvenile, and 250 (72%) adults (Table 1).

**Table 1:** Capybaras captured in nine areas during 2015–2018.

Areas	Captured capybaras			Gender		Age		
	Total No. captures	No. different individuals	No. recaptures (%)	Males	Females	Young	Juvenile	Adult
BSF-endemic areas								
1-Piracicaba	65	48	17 (26)	9	56	3	10	52
2-Americana	23	20	3 (13)	12	11	0	7	16
3-Araras	41	36	5 (12)	7	34	8	7	26
BSF-nonendemic areas								
4-Pirassununga-A	26	22	4 (15)	10	16	1	4	21
5-Pirassununga-B	73	68	5 (7)	19	54	8	9	56
6-Ribeirão Preto	48	37	11 (23)	16	32	4	24	20
7-São Paulo	14	14	0 (0)	4	10	2	1	11
Natural areas								
8-Poconé	26	13	13 (50)	9	17	0	6	20
9-Corumbá	31	29	2 (6)	8	23	1	2	28
TOTAL	347	287	60 (17)	94 (27%)	253 (73%)	27 (7.8%)	70 (20.2%)	250 (72.0%)

### Serology of capybaras

**Fig 2:**
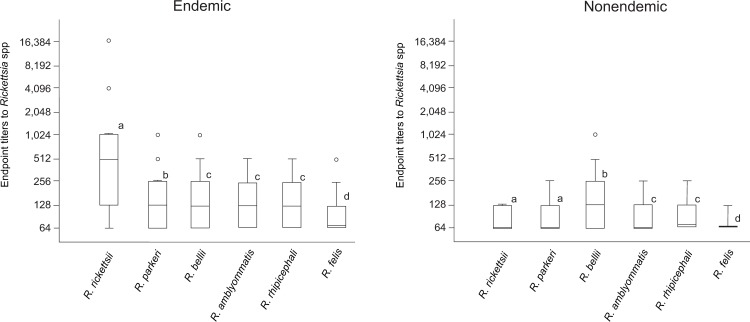
Boxplot representing the serological endpoint titers for six *Rickettsia* species of capybaras from Brazilian spotted fever (BSF)-endemic areas and BSF-nonendemic areas. Different lower case letters mean statistically different (*P*<0.05) endpoint titers between *Rickettsia* species in endemic or nonendemic areas.

Among the 347 captured capybaras, sera were collected from 337, which were tested by IFA against six *Rickettsia* species (Table 2). Considering the three epidemiological categories, the proportions of seropositive capybaras for *R*. *rickettsii* in the 3 BSF-endemic areas (88 to 98%) were significantly higher (*P*<0.05) than the proportions in the 4 BSF-nonendemic areas (14 to 38%) and in the natural area of Corumbá (47%); the proportions for *R*. *rickettsii* in the later 5 areas were statistically similar (*P*>0.05). While the proportion of seropositive capybaras in the natural area of Poconé (100%) was similar (*P*>0.05) to the BSF-endemic areas, the endpoint titers to *R*. *rickettsii* were quite different, with much higher values for the BSF-endemic areas (S2 Table). In fact, 56 capybaras of the 3 BSF-endemic areas had endpoint titers for *R*. *rickettsii* at least fourfold higher than the titers for the remaining five *Rickettsia* species, indicating that these capybaras were likely infected by *R*. *rickettsii* (Table 2). Using these same criteria, no capybara from either BSF-nonendemic or natural areas were considered to have been infected by *R*. *rickettsii*, whereas 12, 36, and 11 capybaras from BSF-endemic, BSF-nonendemic, and natural areas, respectively, were probably infected by *R*. *bellii*; and two, one and nine capybaras from BSF-endemic, BSF-nonendemic, and natural areas, respectively, were probably infected by *R*. *amblyommatis*. In addition, five capybaras from the natural area of Poconé were likely infected by *R*. *parkeri* (Table 2). While the endpoint titers of the capybaras from the BSF-endemic areas were significantly higher for *R*. *rickettsii* than for the remaining five *Rickettsia* species, in the BSF-nonendemic areas the endpoint titers were significantly higher for *R*. *bellii* (Fig 2).

**Table 2:** Results of immunofluorescence assay for six *Rickettsia* species in capybaras from 9 localities, being 3 Brazilian spotted fever (BSF)-endemic areas, 4 BSF-nonendemic areas, and 2 natural areas.

Areas	No. capybaras tested	No. seroreactive capybaras to each of the *Rickettsia* species (% seroreactivity for the area)						No. capybaras with determined homologous reaction (PAIHR in parentheses) ^*a*^
		*Rickettsia rickettsii* ^*b*^	*Rickettsia parkeri*	*Rickettsia amblyommatis*	*Rickettsia rhipicephali*	*Rickettsia bellii*	*Rickettsia felis*	
BSF-endemic areas								
1-Piracicaba	64	63 (98) a	62 (97)	56 (87)	55 (86)	54 (84)	27 (42)	35 (*R*. *rickettsii*),4 (*R*. *bellii*), 2 (*R*. *amblyommatis*)
2-Americana	23	22 (97) a	21 (91)	15 (65)	14 (61)	20 (87)	11 (48)	8 (*R*. *rickettsii*), 1 (*R*. *bellii*)
3-Araras	33	29 (88) a	25 (76)	18 (55)	19 (58)	24 (73)	9 (27)	13 (*R*. *rickettsii*), 7 (*R*. *bellii*)
BSF-nonendemic areas								
4-Pirassununga-A	26	10 (38) b	8 (31)	9 (35)	10 (38)	14 (54)	6 (23)	9 (*R*. *bellii*)
5-Pirassununga-B	73	26 (36) b	14 (19)	11 (15)	13 (18)	32 (44)	10 (14)	11 (*R*. *bellii*)
6-Ribeirão Preto	48	14 (29) b	9 (19)	13 (27)	9 (19)	25 (52)	4 (8)	10 (*R*. *bellii*), 1 (*R*. *amblyommatis*)
7-São Paulo	14	2 (14) b	1 (7)	2 (14)	4 (26)	7 (50)	0 (0)	6 (*R*. *bellii*)
Natural areas								
8-Poconé	26	26 (100) a	26 (100)	24 (92)	23 (88)	25 (96)	20 (77)	7 (*R*. *amblyommatis*), 5 (*R*. *parkeri*), 1 (*R*. *bellii*)
9-Corumbá	30	14 (47) b	11 (37)	11 (37)	7 (23)	17 (57)	0 (0)	10 (*R*. *bellii*), 2 (*R*. *amblyommatis*)

^*a*^ homologous reaction was determined when an endpoint titer to a *Rickettsia* species was at least 4-fold higher than those observed for the other *Rickettsia* species. In this case, the *Rickettsia* species involved in the highest endpoint titer was considered the possible antigen involved in a homologous reaction (PAIHR).

^*b*^ different letters in this column mean significantly different (*P*<0.05) proportions of seroreactive cabybaras for *R*. *rickettsii*.

### Ticks on capybaras

**Fig 3:**
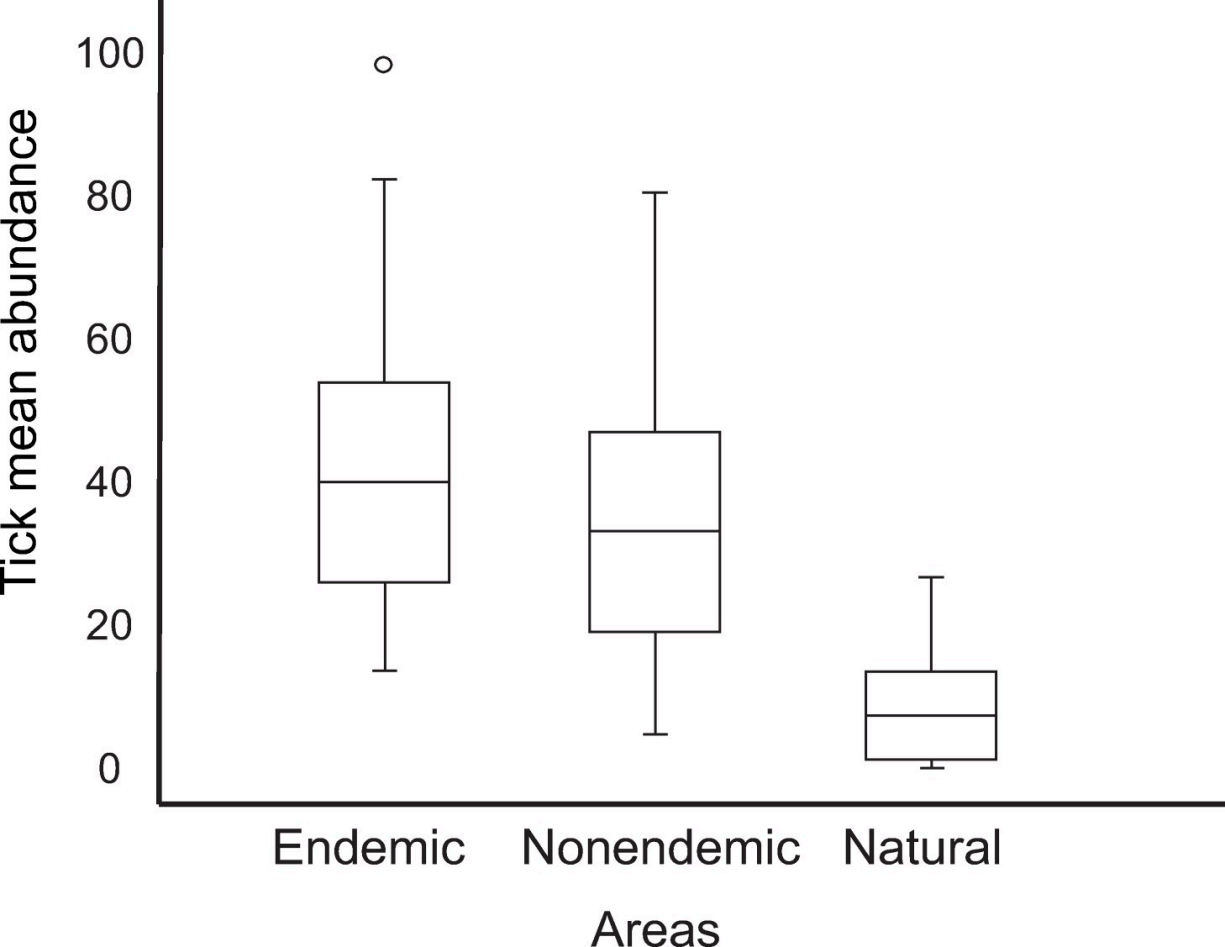
Boxplot representing the mean abundance of total tick infestations of capybaras from Brazilian spotted fever (BSF)-endemic areas, BSF-nonendemic areas, and natural areas.

**Fig 4:**
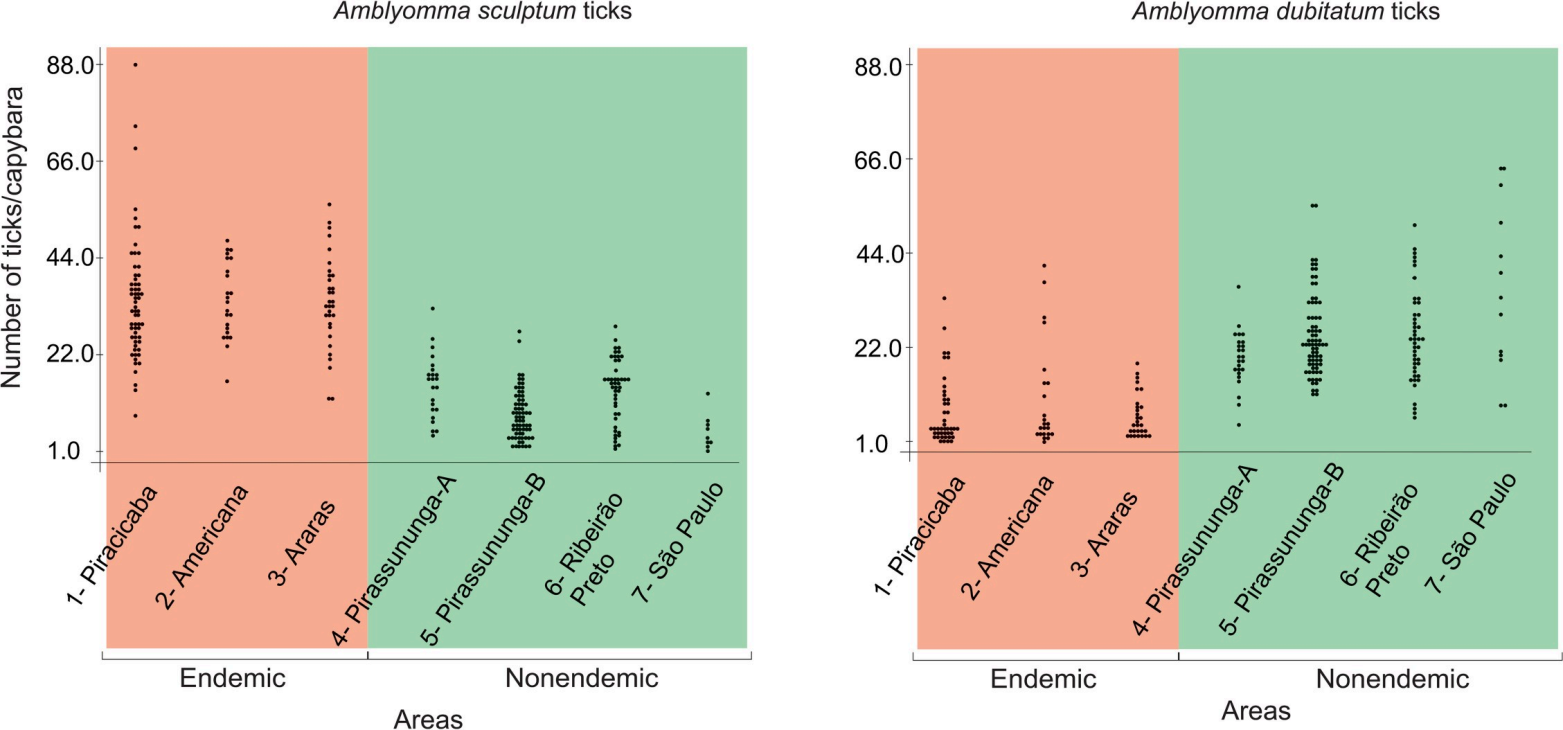
Dotplot representing the number of *Amblyomma sculptum* and *Amblyomma dubitatum* ticks per capybara among 3 Brazilian spotted fever (BSF)-endemic areas, and 4 BSF-nonendemic areas.

**Fig 5:**
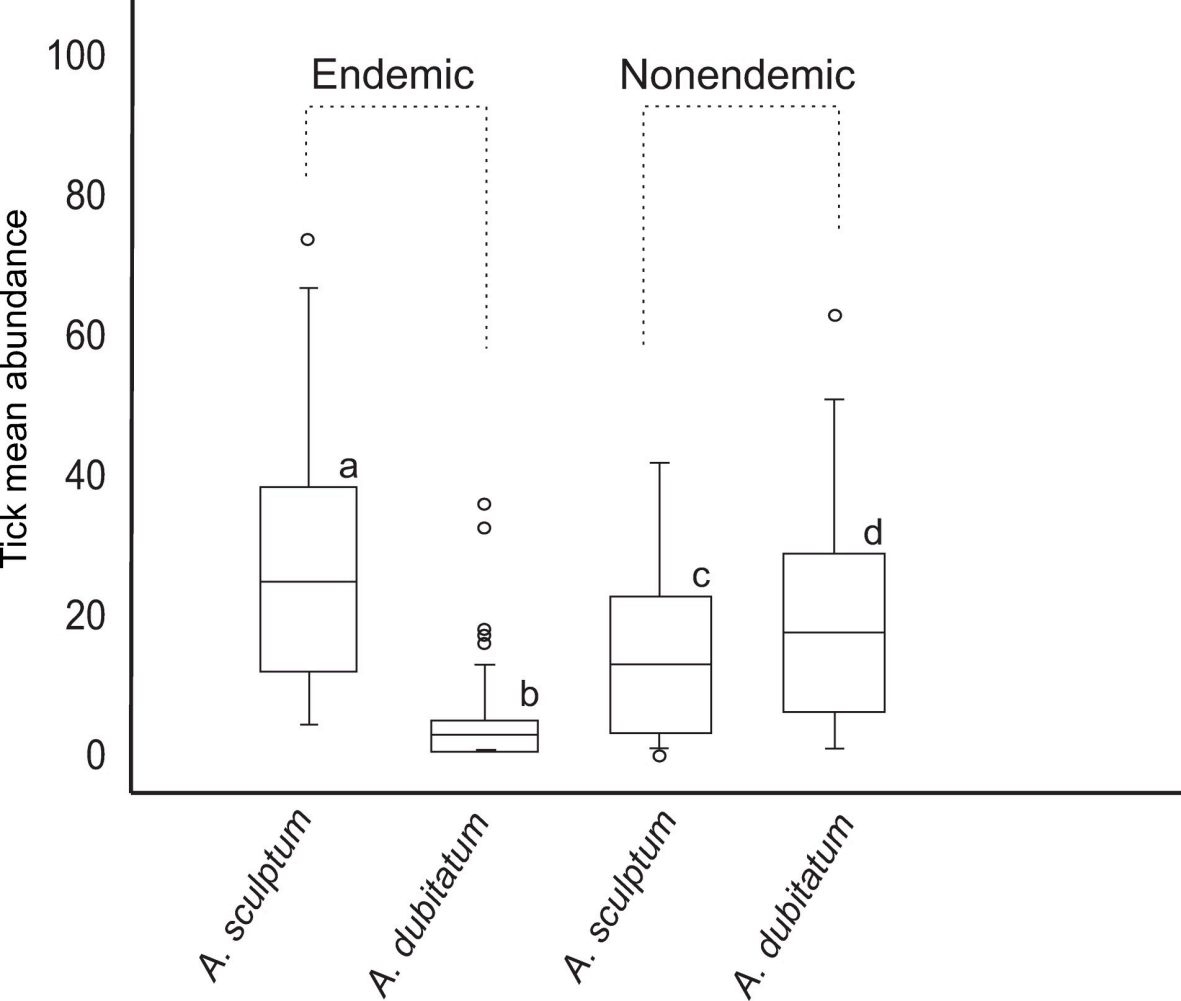
Boxplot representing the mean abundance of *Amblyomma sculptum* and *Amblyomma dubitatum* infestations of capybaras from Brazilian spotted fever (BSF)-endemic areas and BSF-nonendemic areas. Different lower case letters mean statistically different (*P*<0.05) mean abundance values of either *A*. *sculptum* or *A*. *dubitatum* between endemic and nonendemic areas.

Capybaras in the seven anthropic areas of the state of São Paulo (BSF-endemic and BSF-nonendemic areas) were infested by two tick species, *A*. *sculptum* and *A*. *dubitatum*. Among the two natural areas in the Pantanal biome, *A*. *sculptum* was the only species infesting capybaras in the Corumbá area, while *A*. *sculptum*, *A*. *dubitatum* and *Amblyomma triste* were found on capybaras in the Poconé area. Tick prevalence on capybaras was 100% in all seven anthropic areas, and 95% in natural areas, where only three capybaras did not have any tick. For data comparison, we excluded the 27 young capybaras (Table 1) because they were usually infested by low number of ticks (mean abundance: 15.3 ticks/capybara), when compared to the overall mean abundance of 31.8 ticks/capybara among adults and juveniles. The overall mean abundance of ticks was significantly higher (*P*<0.05) in the BSF-endemic areas (40.9 ticks/capybara) than in the BSF-nonendemic areas (33.7 ticks/capybara), which was also significantly higher (*P*<0.05) than the mean abundance in the natural areas (7.7 ticks/capybara) (Table 3 and Fig 3). *Amblyomma sculptum* was the dominant tick species in the BSF-endemic areas, where they represented 85% (4,091/4,821) of all ticks collected from capybaras (Table 3) and had the highest mean abundance values (Fig 4). In contrast, *A*. *dubitatum* was the dominant species in the BSF-nonendemic areas, where they encompassed 69% (3390/4914) of all ticks collected on capybaras (Table 3) and had the highest mean abundance values (Fig 4). Mean abundance values of either *A*. *sculptum* or *A*. *dubitatum* were significantly different (*P*<0.05) between BSF-endemic and BSF-nonendemic areas (Fig 5). In the natural areas, *A*. *sculptum* was the dominant tick species (88%; 380/429); however, with a mean abundance of only 6.8 *A*. *sculptum* ticks/capybara, contrasting to the mean abundance of 34.7 and 10.2 *A*. *sculptum* ticks/capybara in the BSF-endemic and BSF-nonendemic areas, respectively.

**Table 3:** Ticks collected on capybaras in 9 localities, being 3 Brazilian spotted fever (BSF)-endemic areas, 4 BSF-nonendemic areas, and 2 natural areas during 2015–2018.

Areas	N	P	MA^*a*^	No. ticks according to species and stage									
				*Amblyomma sculptum*			*Amblyomma dubitatum*			*Amblyomma triste*		*Amblyomma* sp.	Total
				nymphs	females	males	nymphs	females	males	females	males	larvae	
BSF-endemic areas													
1-Piracicaba	62	100	39.7	987	665	491	191	76	32			17	2,459
2-Americana	23	100	44.1	325	216	250	137	60	26			1	1,015
3-Araras	33	100	40.8	705	265	187	114	58	18				1,347
Total	118	100	40.9 a	2,017	1,146	928	442	194	76			18	4,821
BSF-nonendemic areas													
4-Pirassununga-A	25	100	33.7	75	147	106	382	70	43			19	842
5-Pirassununga-B	65	100	30.1	197	145	187	720	369	338				1,956
6-Ribeirão Preto	44	100	37.6	410	110	85	671	239	135			4	1,654
7-São Paulo	12	100	38.5	31	5	3	198	103	122				462
Total	146	100	33.7 b	713	407	381	1,971	781	638			23	4,914
Natural areas													
8-Poconé	26	92	10.9	53	72	109	3	17	18	2	9		283
9-Corumbá	30	97	4.9	88	28	30							146
Total	56	95	7.7 c	141	100	139	3	17	18	2	9		429
Total (9 areas)	320	99	31.8	2,871	1,653	1,448	2,416	992	732	2	9	41	10,164

N: Number of examined capybaras; P: prevalence = No. infested capybaras / No. examined capybaras x 100; MA: mean abundance of tick infestation = total No. collected ticks / No. examined capybaras.

^*a*^ different letters in this column mean significantly different (*P*<0.05) MA values between the three epidemiological categories.

### Host-questing ticks

Host questing ticks were collected along four consecutive years, during the activity peaks of larvae (autumn), nymphs (winter) and adults (summer) of *A*. *sculptum* in each year. In the anthropic area of São Paulo (area no. 7), dragging was performed only at two instances during the first year (2015); thereafter, this area had to be discontinued from the study due to a highly fatal outbreak of fascioliasis that decimated the capybara population of the area [47], what certainly impacted the environmental tick burdens in the subsequent years. In the remaining six anthropic areas of the state of São Paulo (areas no 1 to 6), dragging was not possible only during the adult tick season of the 2016 summer, due to personal problems beyond our control. In the two natural areas (Poconé and Corumbá), dragging was not possible at two occasions in each area due to logistic problems related to road conditions and access to both areas. Overall, dragging was performed at 11 occasions in each of the three BSF-endemic areas, at 11 occasions in two BSF-nonendemic areas (Pirassununga A and Pirassununga B), at 20 occasions in the BSF-nonendemic area of Ribeirão Preto, at 19 occasions in the natural area of Poconé, and at 16 occasions in the natural area of Corumbá.

A total of 21,670 ticks were collected by dragging in all areas during the study. In the anthropic areas, only two tick species were identified, *A*. *sculptum* and *A*. *dubitatum*. In the natural areas, the following six tick species were collected: *A*. *sculptum*, *A*. *dubitatum*, *Amblyomma parvum*, *A*. *triste*, *Ambyomma ovale*, and *Ornithodoros rostratus* (Table 4). In the BSF-endemic areas, the proportions of *A*. *sculptum* and *A*. *dubitatum* were 92% (10,425/11,305) and 8% (880/11,305), respectively. In contrast, the proportions of *A*. *sculptum* and *A*. *dubitatum* in the BSF-nonendemic areas were 43% (3,688/8,633) and 57% (4,945/8,633), respectively. These proportions were significantly different (*P*<0.05) between the two epidemiological categories. In the natural areas, *A*. *sculptum* represented 98% (1,694/1,732) of all collected ticks, a proportion significantly different (*P*<0.05) from the two categories of anthropic areas.

**Table 4:** Host-questing ticks collected by dragging during 2015–2019 in 9 localities, being 3 Brazilian spotted fever (BSF)-endemic areas, 4 BSF-nonendemic areas, and 2 natural areas.

Areas	No. ticks according to species and stage											Total
	*Amblyomma sculptum*			*Amblyomma dubitatum*			*Amblyomma parvum*		*Amblyomma triste*	*Amblyomma ovale*	*Ornithodoros rostratus*	
	Larval clusters	nymphs	adults	Larval clusters	nymphs	adults	nymphs	adults	adults	adults	nymphs	
BSF-endemic areas												
1-Piracicaba	131	1,646	887	10	196	49						2,919
2-Americana	119	2,781	1,464	11	288	27						4,690
3-Araras	117	2,576	704	9	261	29						3,696
Total	367	7,003	3,055	30	745	105						11,305
BSF-nonendemic areas												
4-Pirassununga-A	48	461	132	61	276	221						1,199
5-Pirassununga-B	56	1,143	118	81	1,464	151						3,013
6-Ribeirão Preto	159	1,218	342	161	2,143	351						4,374
7-São Paulo		5	6		15	21						47
Total	263	2,827	598	303	3,898	744						8,633
Natural areas												
8-Poconé	48	618	139		2				12	2		821
9-Corumbá	65	672	152				15	2		1	4	911
Total	113	1,290	291		2		15	2	12	3	4	1,732
Total (9 areas)	743	11,120	3,944	333	4,645	849	15	2	12	3	4	21,670

**Fig 6:**
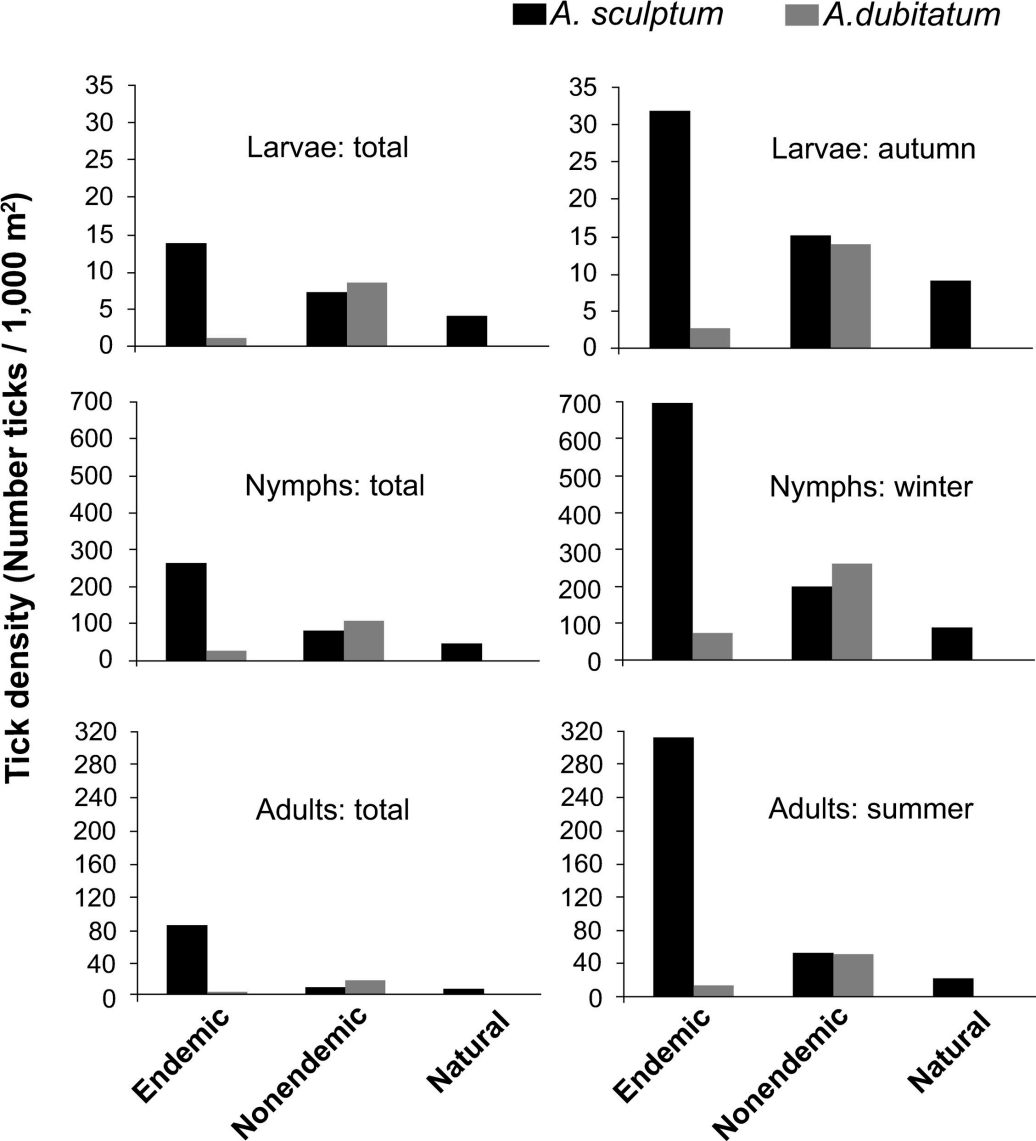
Density of host-questing larvae, nymphs and adult ticks (*Amblyomma sculptum* and *Amblyomma dubitatum*) collected during all seasons of the study period (total) or during the larval (autumn), nymphal (winter) or adult (summer) seasons during 2015–2019 in Brazilian spotted fever (BSF)-endemic areas, BSF-nonendemic areas, and natural areas.

Tick density (TD) values, represented by the number of host-questing ticks per 1,000 m^2^, were calculated for the two most abundant tick species, *A*. *sculptum* and *A*. *dubitatum*, in all areas. Grouping all dragging occasions during the four years, TD of *A*. *sculptum* larvae, nymphs and adults were higher in BSF-endemic areas than in BSF-nonendemic and natural areas, with some significant (*P*<0.05) differences (Table 5 and Fig 6). Comparisons of TD values of *A*. *sculptum* with those of *A*. *dubitatum* revealed significantly higher values (*P*<0.05) for larvae, nymphs and adults of the former species in the BSF-endemic areas. On the other hand, *A*. *sculptum* and *A*. *dubitatum* had similar (*P*>0.05) larval, nymphal and adult TD among the BSF-nonendemic areas (S2 Table). Because only two *A*. *dubitatum* nymphs were collected by dragging in the natural areas (Table 4), TD values were not statistically compared with *A*. *sculptum* in these areas.

**Table 5:** Density of host-questing ticks in 9 localities [3 Brazilian spotted fever (BSF)-endemic areas, 4 BSF-nonendemic areas, and 2 natural areas] during 2015–2019. In each area, dragging was performed up to three times a year, each at autumn, winter and summer seasons.

Areas	Total area dragged (m^2^)	Tick density (per 1,000 m^2^) ^*a*^					
		*Amblyomma sculptum*			*Amblyomma dubitatum*		
		Adults	nymphs	Larvae	Adults	Nymphs	Larvae
BSF-endemic areas							
1-Piracicaba	8,800	100.8	187.1	14.9	5.6	22.3	1.1
2-Americana	8,800	166.4	316.0	13.5	3.1	32.7	1.3
3-Araras	8,800	80.0	292.7	13.3	3.3	29.7	1.0
Total	**26,400**	**115.7** ^**A,a**^	**265.3** ^**A,a**^	**13.9** ^**A,a**^	**4.0** ^**A,b**^	**28.2** ^**A,b**^	**1.1** ^**A,b**^
BSF-nonendemic areas							
4-Pirassununga-A	8,800	15.0	52.4	5.5	25.1	31.4	6.9
5-Pirassununga-B	8,800	13.4	129.9	6.4	17.2	166.4	9.2
6-Ribeirão Preto	16,000	21.4	76.1	9.9	21.9	133.9	10.1
7-São Paulo	1,600	3.8	3.1	0.0	13.1	9.4	0.0
Total	**35,200**	**17.0** ^**B,a**^	**80.3** ^**A,a**^	**7.5** ^**A,a**^	**21.1** ^**B,a**^	**110.4** ^**B,a**^	**8.6** ^**B,a**^
Natural areas							
8-Poconé	15,200	9.1	40.7	3.2	0	0.1	0
9-Corumbá	12,800	11.9	52.5	5.1			
Total	**28,000**	**10.4** ^**B**^	**46.1** ^**A**^	**4.0** ^**B**^	**0**	**0.1**	**0**

^*a*^ different capital letters in each column mean significantly different (*P*<0.05) tick density values between the three epidemiological categories (BSF-endemic areas, BSF-nonendemic areas, Natural area); different lowercase letters in each line mean significantly different (*P*<0.05) tick density values of adults or nymphs or larvae between *A*. *sculptum* and *A*. *dubitatum*.

Grouping the dragging occasions that were performed during only the autumn season (larval peak) of the four years, larval TD of *A*. *sculptum* in the BSF-endemic areas (31.8 larval clusters/1,000 m^2^) was ≈2 times higher (*P*<0.05) than in the BSF-nonendemic areas (15.1 larval clusters/1,000 m^2^), and ≈3 times higher (*P*<0.05) than in the natural areas (9.1 larval clusters/1,000 m^2^). On the other hand, larval TD of *A*. *dubitatum* in the BSF-endemic areas (2.7 larval clusters/1,000 m^2^) was about ≈4 times lower (*P*<0.05) than in the BSF-nonendemic areas (13.8 larval clusters/1,000 m^2^) (Table 6 and Fig 6).

**Table 6:** Density of host-questing ticks in 9 localities [3 Brazilian spotted fever (BSF)-endemic areas, 4 BSF-nonendemic areas, and 2 natural areas] for ticks collected only during autumn (May or June) of the years 2015–2018.

Areas	Total area dragged (m^2^)	Tick density per 1,000 m^2^					
		*Amblyomma sculptum*			*Amblyomma dubitatum*		
		Adults	nymphs	Larvae	Adults	Nymphs	Larvae
BSF-endemic areas							
1-Piracicaba	3,200	10.9	5.9	35.9	0.3	4.1	2.8
2-Americana	3,200	0	15.6	29.7	0	1.3	3.1
3-Araras	3,200	0.3	5.6	29.7	0	0.6	2.2
Total	**9,600**	**3.8**	**9.1**	**31.8** ^**A,a**^	**0.1**	**2.0**	**2.7** ^**A,b**^
BSF-nonendemic areas							
4-Pirassununga-A	3,200	0	3.1	10.9	4.7	6.3	12.2
5-Pirassununga-B	3,200	0	5.6	11.9	0	18.8	15.9
6-Ribeirão Preto	4,800	1.3	3.1	22.5	1.3	15.8	15.6
7-São Paulo	800	5.0	3.8	0	11.3	13.8	0
Total	**12,000**	**0.8**	**3.8**	**15.1** ^**B,a**^	**2.5**	**13.9**	**13.8** ^**B,a**^
Natural areas							
8-Poconé	4,000	1.3	2.8	3.0	0	0	0
9-Corumbá	1,600	11.9	17.5	24.4			
Total	**5,600**	**4.3**	**7.0**	**9.1** ^**C**^	**0**	**0**	**0**

^*a*^ different capital letters in each column mean significantly different (*P*<0.05) tick density values of larvae between the three epidemiological categories (BSF-endemic areas, BSF-nonendemic areas, Natural area); different lowercase letters in each line mean significantly different (*P*<0.05) tick density values of larvae between *A*. *sculptum* and *A*. *dubitatum*.

During the winter season (nymphal peak) of the four years, nymphal TD of *A*. *sculptum* in the BSF-endemic areas (703.9 nymphs/1,000 m^2^) was ≈3.5 times higher (*P*<0.05) than in the BSF-nonendemic areas (199.6 nymphs/1,000 m^2^), and ≈9 times higher (*P*<0.05) than in the natural areas (86.7 nymphs/1,000 m^2^). On the other hand, nymphal TD of *A*. *dubitatum* in the BSF-endemic areas (72.1 nymphs/1,000 m^2^) was about ≈3.5 times lower (*P*<0.05) than in the BSF-nonendemic areas (262.3 nymphs/1,000 m^2^) (Table 7 and Fig 6).

**Table 7:** Density of host-questing ticks in 9 localities [3 Brazilian spotted fever (BSF)-endemic areas, 4 BSF-nonendemic areas, and 2 natural areas] for ticks collected only during winter (August or September) of the years 2015–2018.

Areas	Total area dragged (m^2^)	Tick density per 1,000 m^2^					
		*Amblyomma sculptum*			*Amblyomma dubitatum*		
		Adults	Nymphs	Larvae	Adults	Nymphs	Larvae
BSF-endemic areas							
1-Piracicaba	3,200	85.3	480.9	4.4	5.0	52.5	0.3
2-Americana	3,200	130.3	847.2	7.2	0.3	82.8	0.3
3-Araras	3,200	26.3	783.4	6.6	0.0	80.9	0.6
Total	**9,600**	**80.6**	**703.9** ^**A,a**^	**6.0**	**1.8**	**72.1** ^**A,b**^	**0.4**
BSF-nonendemic areas							
4-Pirassununga-A	3,200	3.8	137.2	4.1	24.1	57.2	3.8
5-Pirassununga-B	3,200	11.9	338.8	5.6	16.3	422.5	5.0
6-Ribeirão Preto	6,400	5.3	185.8	5.6	13.1	316.9	12.5
7-São Paulo	800	2.5	2.5	0.0	15.0	5.0	0.0
Total	**13,600**	**6.3**	**199.6** ^**B,a**^	**4.9**	**16.5**	**262.3** ^**B,a**^	**7.9**
Natural areas							
8-Poconé	6,400	5.8	86.4	5.6	0	0.3	0
9-Corumbá	6,400	3.0	87.0	1.4			
Total	**12,800**	**4.4**	**86.7** ^**C**^	**3.5**	**0**	**0.3**	**0**

^*a*^ different capital letters in each column mean significantly different (*P*<0.05) tick density values of nymphs between the three epidemiological categories (BSF-endemic areas, BSF-nonendemic areas, Natural area); different lowercase letters in each line mean significantly different (*P*<0.05) tick density values of nymphs between *A*. *sculptum* and *A*. *dubitatum*.

During the summer season (adult peak) of the four years, adult TD of *A*. *sculptum* in the BSF-endemic areas (311.8 adults/1,000 m^2^) was ≈6 times higher (*P*<0.05) than in the BSF-nonendemic areas (52.3 adults/1,000 m^2^), and ≈14 times higher (*P*<0.05) than in the natural areas (22 adults/1,000 m^2^). On the other hand, adult TD of *A*. *dubitatum* in the BSF-endemic areas (12.1 adults/1,000 m^2^) was about ≈4 times lower (*P*<0.05) than in the BSF-nonendemic areas (50.9 adults/1,000 m^2^) (Table 8 and Fig 6).

**Table 8:** Density of host-questing ticks in 9 localities [3 Brazilian spotted fever (BSF)-endemic areas, 4 BSF-nonendemic areas, and 2 natural areas] for ticks collected only during summer (January or February) of the years 2015–2019.

Areas	Total area dragged (m^2^)	Tick density per 1,000 m^2^					
		*Amblyomma sculptum*			*Amblyomma dubitatum*		
		Adults	Nymphs	Larvae	Adults	Nymphs	Larvae
BSF-endemic areas							
1-Piracicaba	2,400	241.3	36.7	0.8	13.3	6.3	0
2-Americana	2,400	436.3	8.3	0.4	10.8	7.9	4.6
3-Araras	2,400	257.9	21.3	0.4	12.1	0.0	0
Total	**7,200**	**311.8** ^**A,a**^	**22.1**	**0.6**	**12.1**^**A,b**^	**4.7**	**1.5**
BSF-nonendemic areas							
4-Pirassununga-A	2,400	50.0	5.0	0	53.8	30.4	4.2
5-Pirassununga-B	2,400	33.3	17.1	0	41.3	21.7	5.8
6-Ribeirão Preto	4,800	62.9	2.9	3.1	54.4	8.1	1.3
7-São Paulo	Not done						
Total	**9,600**	**52.3** ^**B,a**^	**7.0**	**1.6**	**50.9** ^**B,a**^	**17.1**	**3.1**
Natural areas							
8-Poconé	4,800	20.2	11.3	0	0	0	0
9-Corumbá	4,800	23.8	18.1	3.5			
Total	**9,600**	**22.0** ^**C**^	**14.7**	**1.8**	**0**	**0**	**0**

^*a*^ different capital letters in each column mean significantly different (*P*<0.05) tick density values of adults between the three epidemiological categories (BSF-endemic areas, BSF-nonendemic areas, Natural area); different lowercase letters in each line mean significantly different (*P*<0.05) tick density values of adults between *A*. *sculptum* and *A*. *dubitatum*.

During autumn, winter and summer, TD values of larvae, nymphs and adults, respectively, were always higher (*P*<0.05) for *A sculptum* than for *A*. *dubitatum* in the BSF-endemic areas, but at the same time similar (*P*>0.05) between the two tick species in the BSF-nonendemic areas (Tables 6–8).

A total of 8,790 ticks were collected by 220 dry ice traps during August 2015 in all areas of the study (S3 Table). In the BSF-endemic areas, the mean number of *A*. *sculptum* ticks per trap was 35.4, ≈3.5 times higher than the mean number of *A*. *dubitatum* ticks per trap (9.4). In the BSF-nonendemic areas, the mean numbers of *A*. *sculptum* and *A*. *dubitatum* per trap were similar, 25.2 and 23.8, respectively. In the natural areas, we collected on average 6.3 *A*. *sculptum*/trap and 0.3 *A*. *dubitatum*/trap, in addition to two other species, *A*. *parvum* (0.1 ticks/trap) and *O*. *rostratus* (0.2 ticks/trap).

### Rickettsial detection in ticks

A total of 216 host-questing adults of *A*. *sculptum* [24 from each of 8 sampled areas (ticks from the BSF-nonendemic area of São Paulo were not included)] were tested individually for the presence of rickettsial DNA, but none of them contained rickettsia. On the other hand, rickettsial DNA was successfully amplified in 4 (29%) out of 14 *A*. *parvum* ticks from Corumbá, and in 2 (17%) out of 12 *A*. *triste* from Poconé. The rickettsial DNA amplified from all four *A*. *parvum* ticks was identified as ‘*Candidatus* Rickettsia andeanae’; i.e., their *gltA* and *ompA* partial sequences were 100% identical to the corresponding sequences of this agent in GenBank (KF030931 and KF030932, respectively). The *gltA* and *ompA* partial sequences generated from the two *A*. *triste* ticks were 100% identical to the corresponding sequences of *R*. *parkeri* strain Portsmouth (CP003341). Tick mitochondrial 16S rRNA gene-DNA was successfully amplified from all *Rickettsia-*negative samples, validating our PCR-negative results.

## Discussion

A four-year field evaluation demonstrated marked differences of capybara and environmental tick burdens between the three epidemiological classifications of the sampled areas, namely BSF-endemic, BSF-nonendemic, and natural areas. Among the nine sampled areas, only three were classified as BSF-endemic, based primarily on recent records of human cases of the disease (S1 Table). In order to certify on the presence/absence of *R*. *rickettsii* circulation between capybaras and ticks in all nine study areas, we performed serological analyses of capybaras against antigens of the most frequent *Rickettsia* species that have been reported in Brazil. While cross-reactive antibodies between *Rickettsia* species are often observed, testing a vertebrate serum against the possible *Rickettsia* species known to occur in a given area is ideal because often homologous antibody titers are higher than heterologous antibody titers. In some cases, the differences in titers may be great enough (≥ fourfold higher) to differentiate among the rickettsial species potentially stimulating the immune response [33, 48]. Based on these criteria, the BSF endemic status of areas no. 1 to 3 (Piracicaba, Americana, and Araras) was corroborated by endpoint titers at least fourfold higher for *R*. *rickettsii* than for other *Rickettsia* species in many of the tested capybaras. In fact, we have just reported a successful isolation of *R*. *rickettsii* from *A*. *sculptum* ticks that were parasitizing one of the capybaras that were captured in the BSF-endemic area of Piracicaba, corroborating local circulation of *R*. *rickettsii* between ticks and capybaras [10].

As expected, we had no serological evidence of *R*. *rickettsii* infection in the four BSF-nonendemic areas of São Paulo state. Actually, what we observed in these areas was serological evidence of other *Rickettsia* species, especially *R*. *bellii*. This result should be related to the predominance of *A*. *dubitatum* ticks in these areas, since it has been reported that most of the *A*. *dubitatum* populations are infected by *R*. *bellii* (usually at high infection rates) in multiple areas in the state of São Paulo, including some of the present study [40, 44, 49, 50].

Similarly to the BSF-nonendemic areas, we did not find serological evidence of *R*. *rickettsii* circulation in the two natural areas; however, it was interesting to note that 100% of the capybaras from Poconé were seroreactive to both *R*. *rickettsii* and *R*. *parkeri*, with endpoint titers generally higher for the later. Our findings of *R*. *parkeri-*infected *A*. *triste* ticks in Poconé supports the serological evidence that some of the capybaras from this area have been infected by *R*. *parkeri*, since *A*. *triste* ticks were found infesting capybaras in that area. Finally, the few serological evidence of capybara exposure to *R*. *amblyommatis* could be related to the recent reports of *R*. *amblyommatis* infecting *A*. *sculptum* ticks [38, 51], including the Poconé area [52], where we found seven capybaras with endpoint titers fourfold higher for *R*. *amblyommatis*.

Our tick surveys clearly demonstrated that the BSF-endemic areas were characterized by tick burdens much higher than in the BSF-nonendemic areas, with *A*. *sculptum* encompassing the vast majority of the ticks on either capybaras or in the environment. In contrast, there was a predominance of *A*. *dubitatum* over *A*. *sculptum* in the BSF-nonendemic areas. Considering that both BSF-endemic and BSF-nonendemic areas had similar landscapes, one of the reasons driving the two distinct tick scenarios could be the size of the capybara population of each area. This hypothesis relies on a recent study performed within another highly anthropic area of the state of São Paulo, which in 2006 was not endemic for BSF, had 78 capybaras, and dry ice traps captured a mean of 0.7 *A*. *sculptum*/trap and 3.3 *A*. *dubitatum*/trap; in 2012, the same area had become endemic for BSF, had 230 capybaras (≈3 times higher than in 2006), and dry ice traps captured a mean of 33 *A*. *sculptum*/trap (≈47 times higher than in 2006) and 2.1 *A*. *dubitatum*/trap (≈0.3 times lower than in 2006) [53]. The authors concluded that the emergence of BSF in the area in 2012 was a consequence of the increase of the local capybara population, which in turn, provided the increment of the *A*. *sculptum* population. Unfortunately, the numbers of capybaras among the BSF-endemic and BSF-nonendemic areas were not available for comparisons during the present study. Indeed, further studies should be done in order to verify capybara demographic differences among the areas here prospected. Moreover, these studies should also focus on the reproduction rates of capybara groups, since recent mathematical models have proposed that the establishment of *R*. *rickettsii* in a capybara-sustained *A*. *sculptum* population is dependent on a high reproduction rate of this host species [11, 54].

The predominance of *A*. *dubitatum* over *A*. *sculptum* could also have direct implications on the absence of *R*. *rickettsii* in BSF-non endemic areas, especially because populations of *A*. *dubitatum* have been found naturally infected by *R*. *bellii* throughout the state of São Paulo, usually at high infection rates [40, 44, 49, 50]. One study showed that *R*. *bellii-*infected *A*. *dubitatum* ticks were partially refractory to *R*. *rickettsii*, and were not competent to pass *R*. *rickettsii* transovarially [55]. Thus, as long as *A*. *dubitatum* prevails in one area, *R*. *rickettsii* might not be able to establish an infection in either *A*. *dubitatum* or *A*. *sculptum*. In the case of *A*. *sculptum*, our results and the study of [53] showed that *R*. *rickettsii* was established only when there was an overgrowth population of *A*. *sculptum*, possibly because the proportion of *R*. *rickettsii-*infected *A*. *sculptum* ticks under natural conditions is always very low (<1%) [7–10]. Such assumption might allow us to speculate that any intervention resulting in a drastic reduction of the *A*. *sculptum* population would eliminate the *R*. *rickettsii* infection from the tick population.

Different from the highly anthropic areas of the state of São Paulo, as much as six tick species were collected in the natural areas of the Pantanal biome. Such species richness was somewhat expected in pristine areas of this biome, where several species of medium- to large-sized mammals act as major hosts for ticks, including *A*. *sculptum* [20, 56–60]. While *A*. *sculptum* was the dominant tick species in the natural areas, tick burdens were much lower than in the anthropic areas. Such findings highlight the ecological disequilibrium of the anthropic areas, where much higher tick burdens were associated to a single major host species, the capybara.

Other factors that could be contributing for the BSF endemic or nonendemic status in *A*. *sculptum-*capybara associated areas in southeastern Brazil are inherent to the *A*. *sculptum* populations, namely their susceptibilities to *R*. *rickettsii* infection. This hypothesis relies on a recent study that compared the susceptibility of *R*. *rickettsii* infection among six populations of *A*. *sculptum* [61]. The authors showed that there were significant differences among the susceptibilities of the six tick populations, and suggested that it could be another factor driving the uneven distribution of *R*. *rickettsii* among the wide distribution of *A*. *sculptum* in southeastern Brazil. However, the mechanisms driving these different susceptibilities are yet to be determined.

The relatively low number of sampled areas (nine) distributed among three contiguous biomes could be considered a main drawback of this study. Indeed, higher number of areas per epidemiological category would imply greater robustness to our results. However, the high similarity of our observations by epidemiological category supports our results. Actually, sampling of areas within three different biomes was chosen for their strong relation with spotted fever core features; capybaras and *A*. *sculptum* ticks. In fact, the endemic and non-endemic areas in São Paulo State were intensely anthropized green areas. Although they might have originally been rainforests or savannahs, their natural phytophysiognomies vanished and now share common environmental features characterized by a water source and low grassy areas with relatively few trees, all adequate for capybaras. Similar natural areas maintaining capybara populations had to be found for the control groups as well. Such pristine areas are non-existent in São Paulo State, and the Pantanal biome was the one that provided the most similar features, for example abundant water source and widespread capybara populations. More importantly, all the nine sampled areas are around the center of the wide range of *A*. *sculptum* [20] and away from the geographic boundaries of this tick species, precluding negative effects of extreme weather on our results for ticks.

## Conclusions

The BSF-endemic areas of the state of São Paulo were characterized by overgrowth populations of *A*. *sculptum* that were sustained chiefly by capybaras, and decreased populations of *A*. *dubitatum*. In contrast, the BSF-nonendemic areas with landscape similar to the endemic areas differed by having lower tick burdens and a slight predominance of *A*. *dubitatum* over *A*.*sculptum*, both sustained chiefly by capybaras. Higher species richness of ticks (six species) was found in the natural areas of Pantanal, although environmental tick burdens were lower than in the anthropic areas of São Paulo. While multiple medium- to large-sized mammals have been pointed out as important hosts for *A*. *sculptum* in the Pantanal, the capybara was the only important host for this tick species in the anthropic areas of the present study. The uneven distribution of the presence of *R*. *rickettsii* infection among *A*. *sculptum* populations in highly anthropic areas of the state of São Paulo could be related to the tick population size and its proportion in relation to sympatric *A*. *dubitatum* populations.

## Supporting information

S1 Text General overview of the nine areas (1 to 9) where capybaras and host-questing ticks were sampled in this study. Satellite images were obtained from Google Earth Pro version 7.3, and the final figure was constructed with the use of Microsoft Power Point 2010, version 14.0.7232.5000.
(PDF)

S1 Table Areas where capybaras and ticks were sampled in the present study. (PDF)

S2 Table Antibody endpoint titers determined by immunofluorescence assays (IFA) against antigens of six *Rickettsia* species in sera of capybaras captured in 9 localities, being 3 Brazilian spotted fever (BSF)-endemic areas, 4 BSF-nonendemic areas, and 2 natural areas of Brazil, during 2015–2018. (PDF)

S3 Table Host-questing ticks collected by dry-ice traps in 9 localities [3 Brazilian spotted fever (BSF)-endemic areas, 4 BSF-nonendemic areas, and 2 natural areas] during 2015–2019. (PDF)
