# Supplementary material for: Epidemiology of capybara-associated Brazilian spotted fever
Source: PLoS Negl Trop Dis. 2019 Sep 6;13(9):e0007734. doi: 10.1371/journal.pntd.0007734 (PMC6750615; doi:10.1371/journal.pntd.0007734)

S1 Text. Overview of the nine areas (1 to 9) sampled in the present study.

1-Piracicaba. Brazilian spotted fever-endemic area (elevation of the sight point: 795 m).
Red circle indicates the area where capybaras and host-questing ticks were sampled in this study.

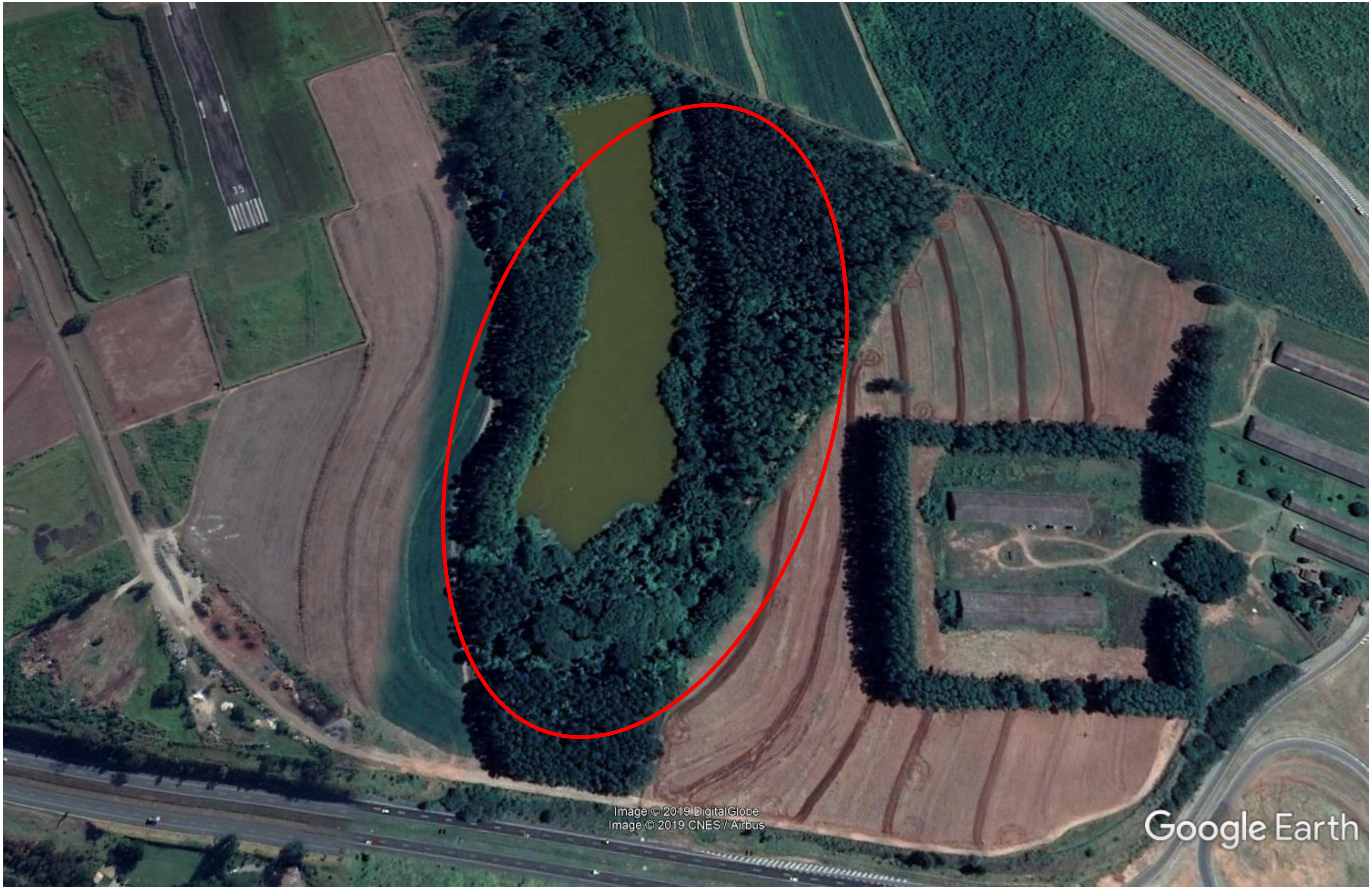

2-Americana. Brazilian spotted fever-endemic area (elevation of the sight point: 780 m).
Red circle indicates the area where capybaras and host-questing ticks were sampled in this study.

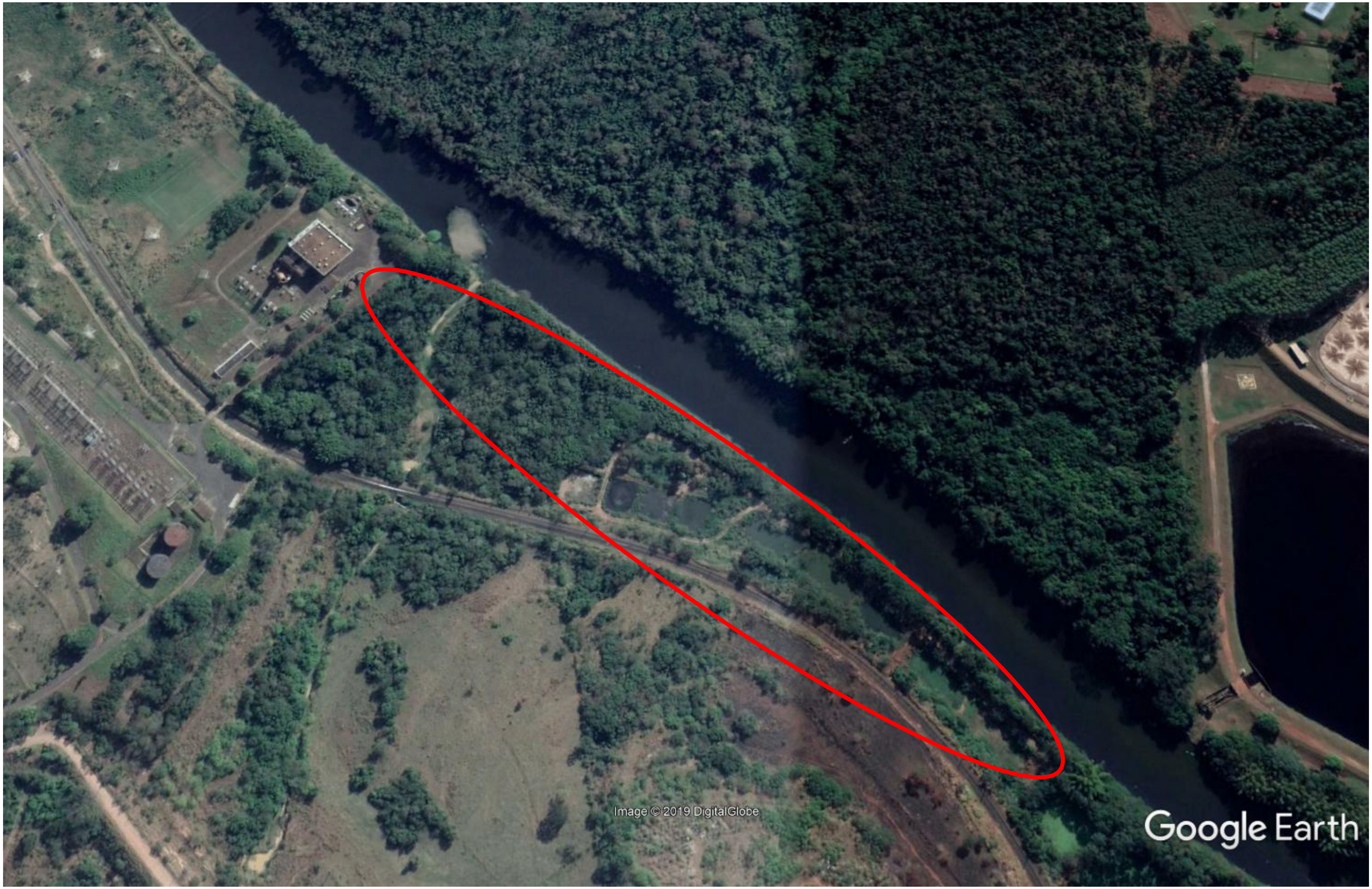

3-Araras. Brazilian spotted fever-endemic area (elevation of the sight point: 887 m).
Red circle indicates the area where capybaras and host-questing ticks were sampled in this study.

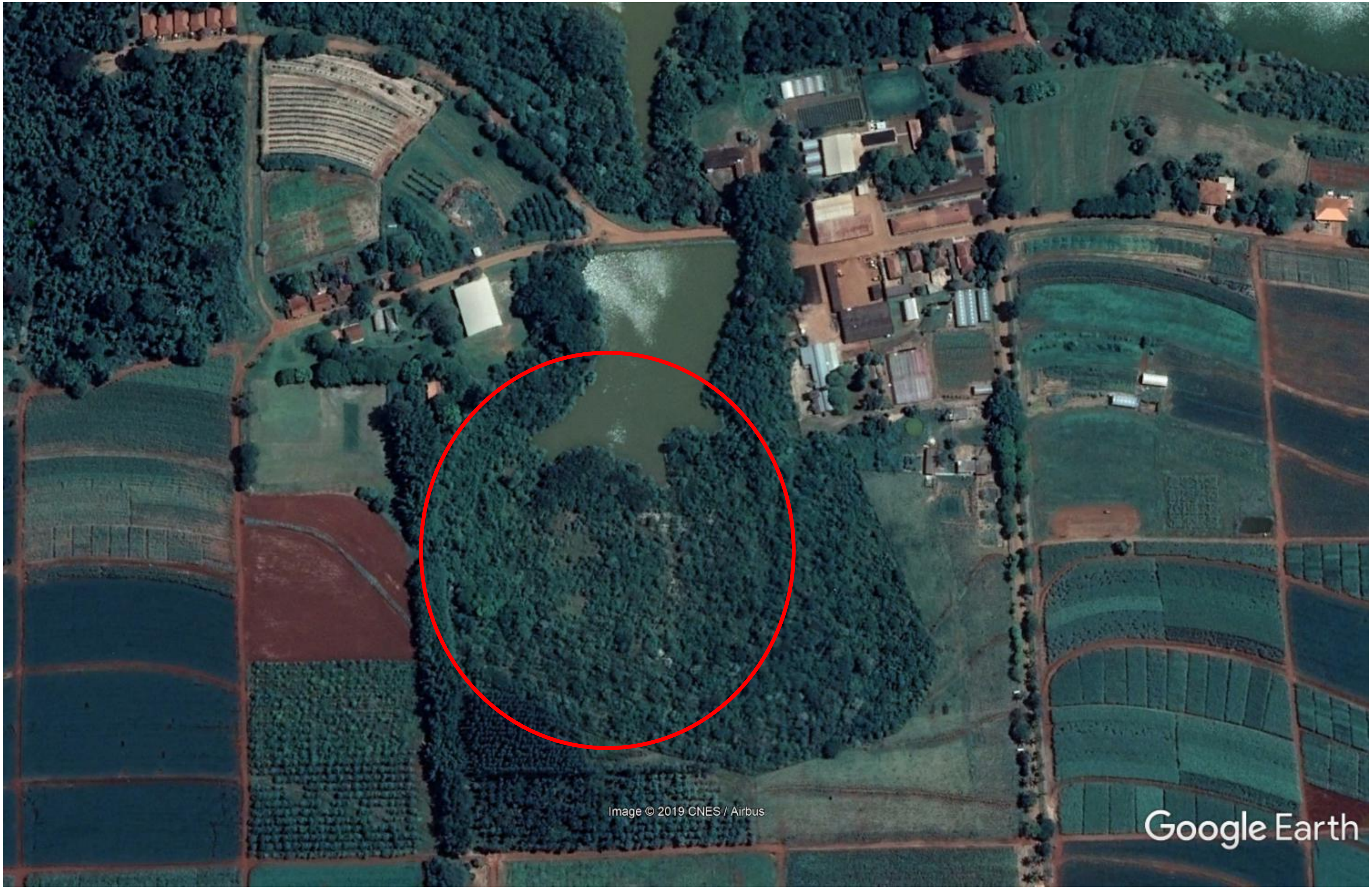

4-Pirassununga A. Brazilian spotted fever-nonendemic area (elevation of the sight point: 850 m). Red circle indicates the area where capybaras and host-questing ticks were sampled in this study.

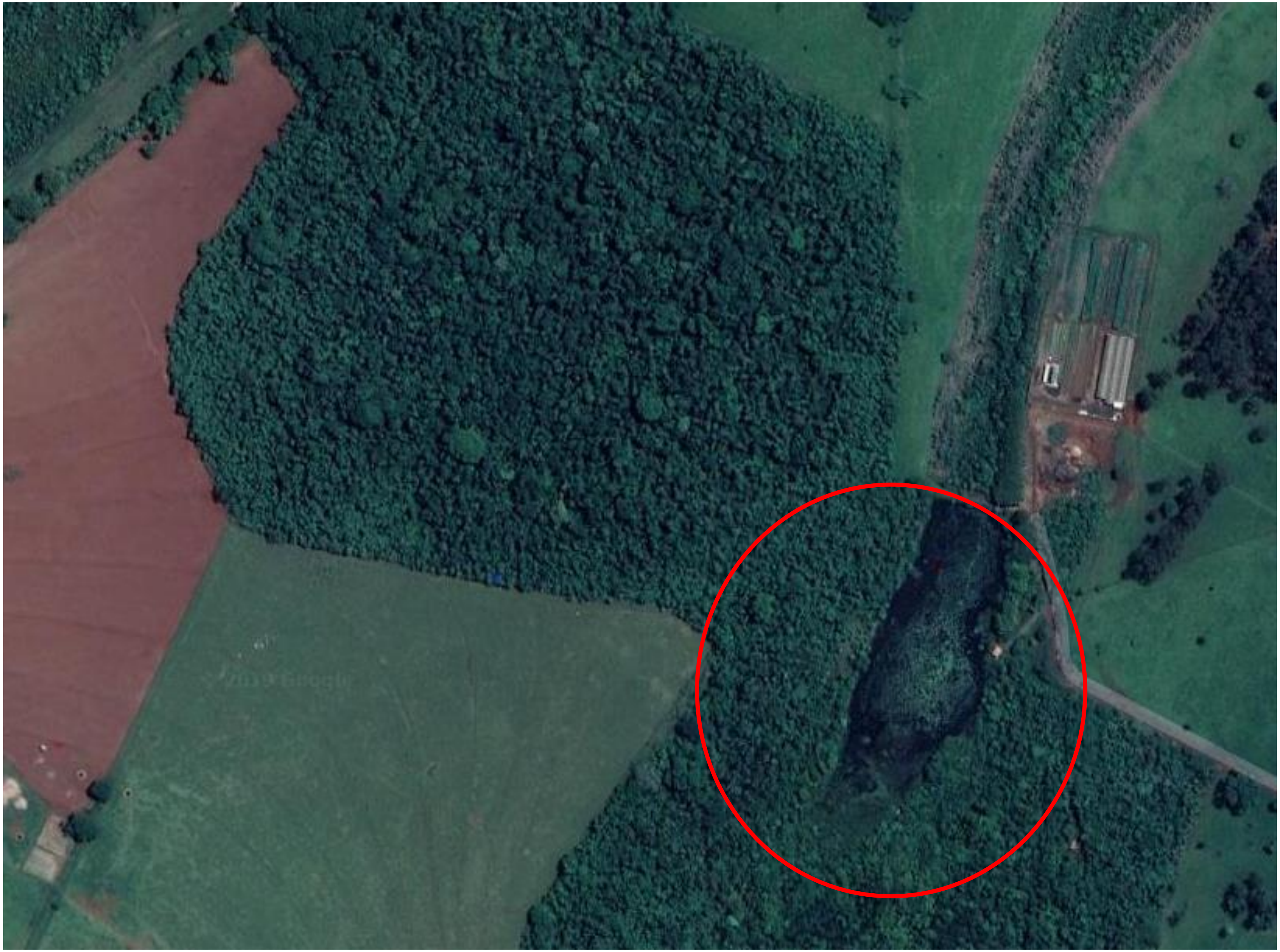

5-Pirassununga B. Brazilian spotted fever-nonendemic area (elevation of the sight point: 842 m). Red circle indicates the area where capybaras and host-questing ticks were sampled in this study.

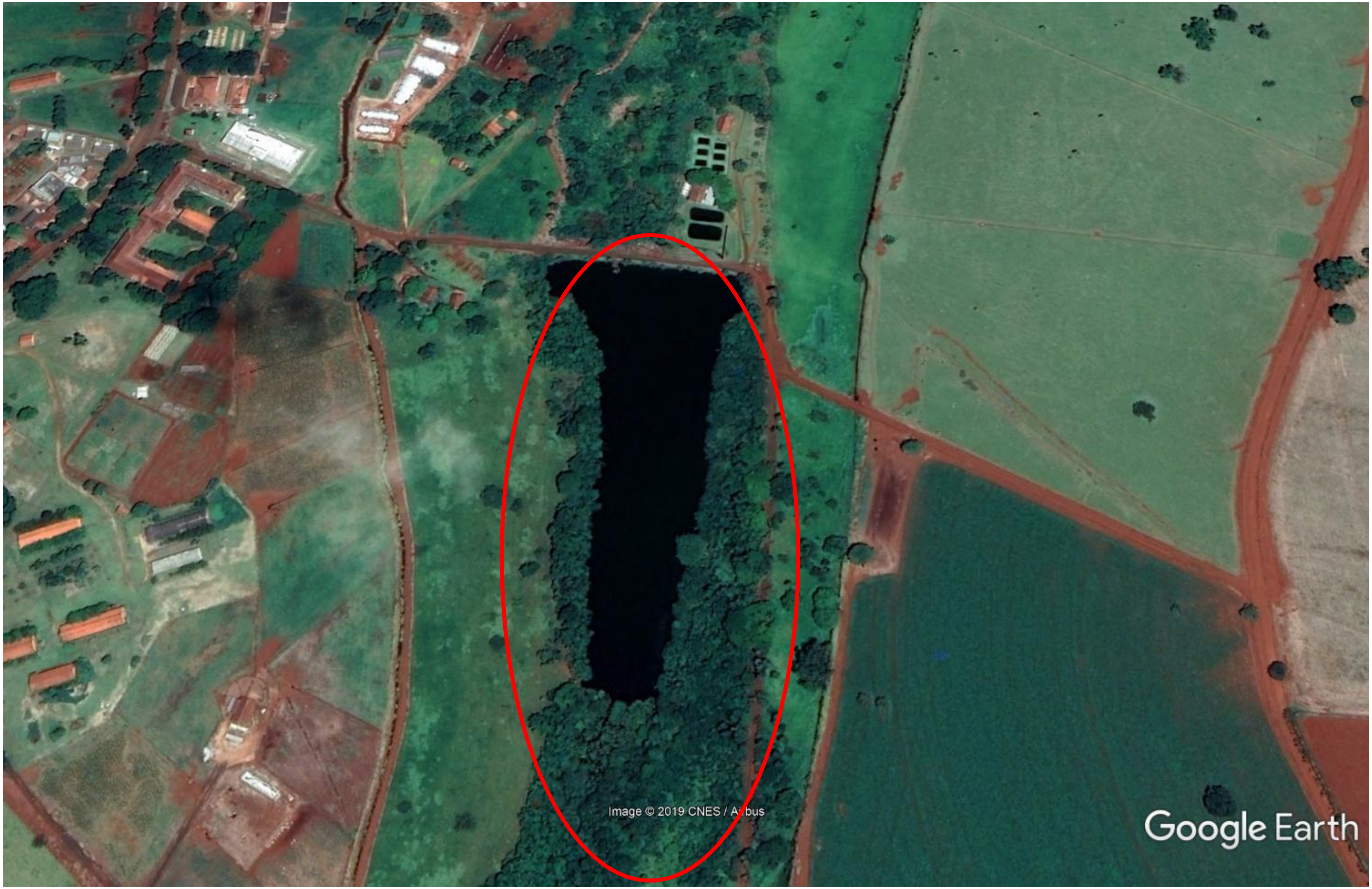

6-Ribeirão Preto. Brazilian spotted fever-nonendemic area (elevation of the sight point: 886 m).
Red circle indicates the area where capybaras and host-questing ticks were sampled in this study.

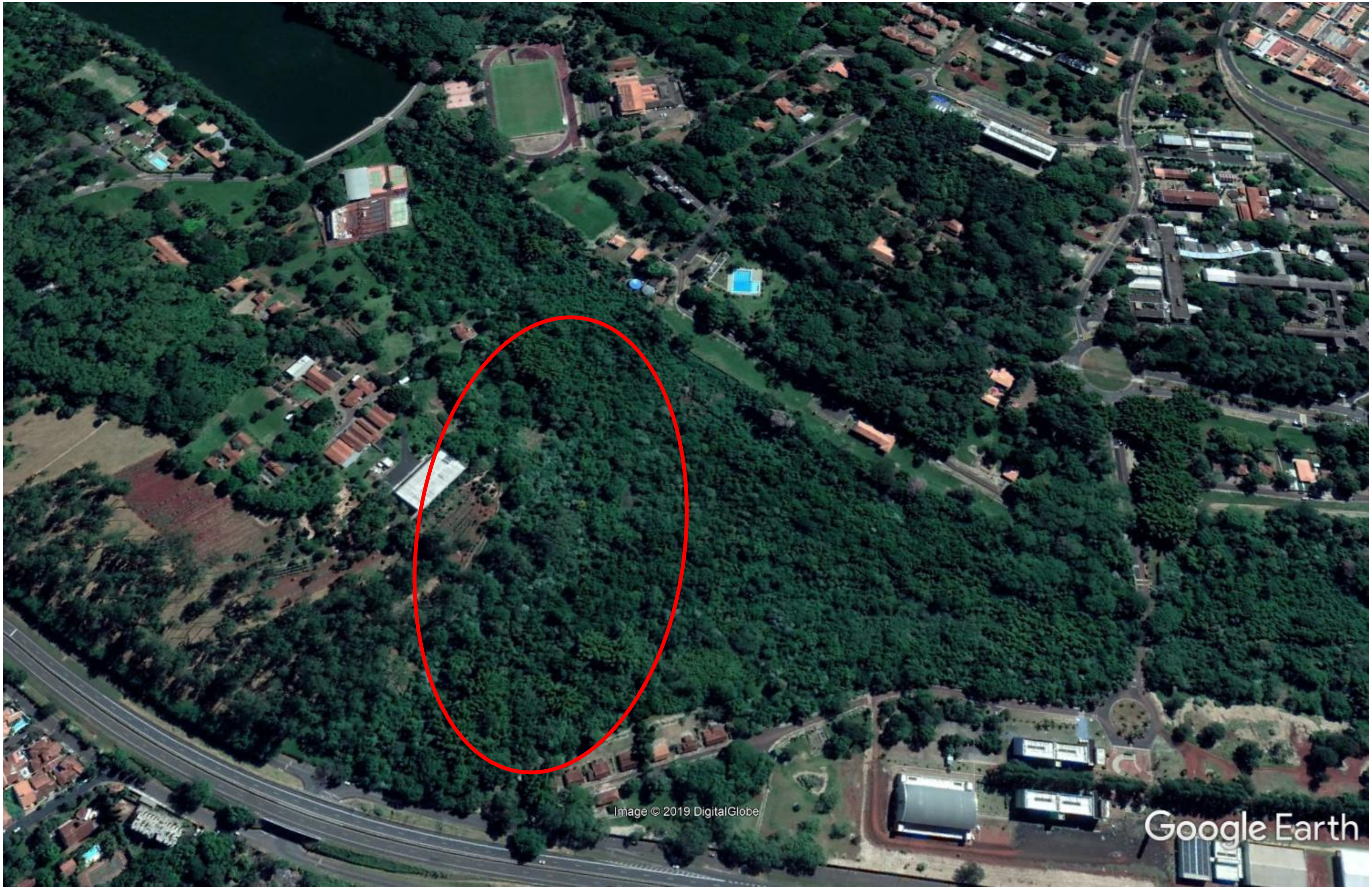

7-São Paulo. Brazilian spotted fever-nonendemic area (elevation of the sight point: 827 m).
Red circle indicates the area where capybaras and host-questing ticks were sampled in this study.

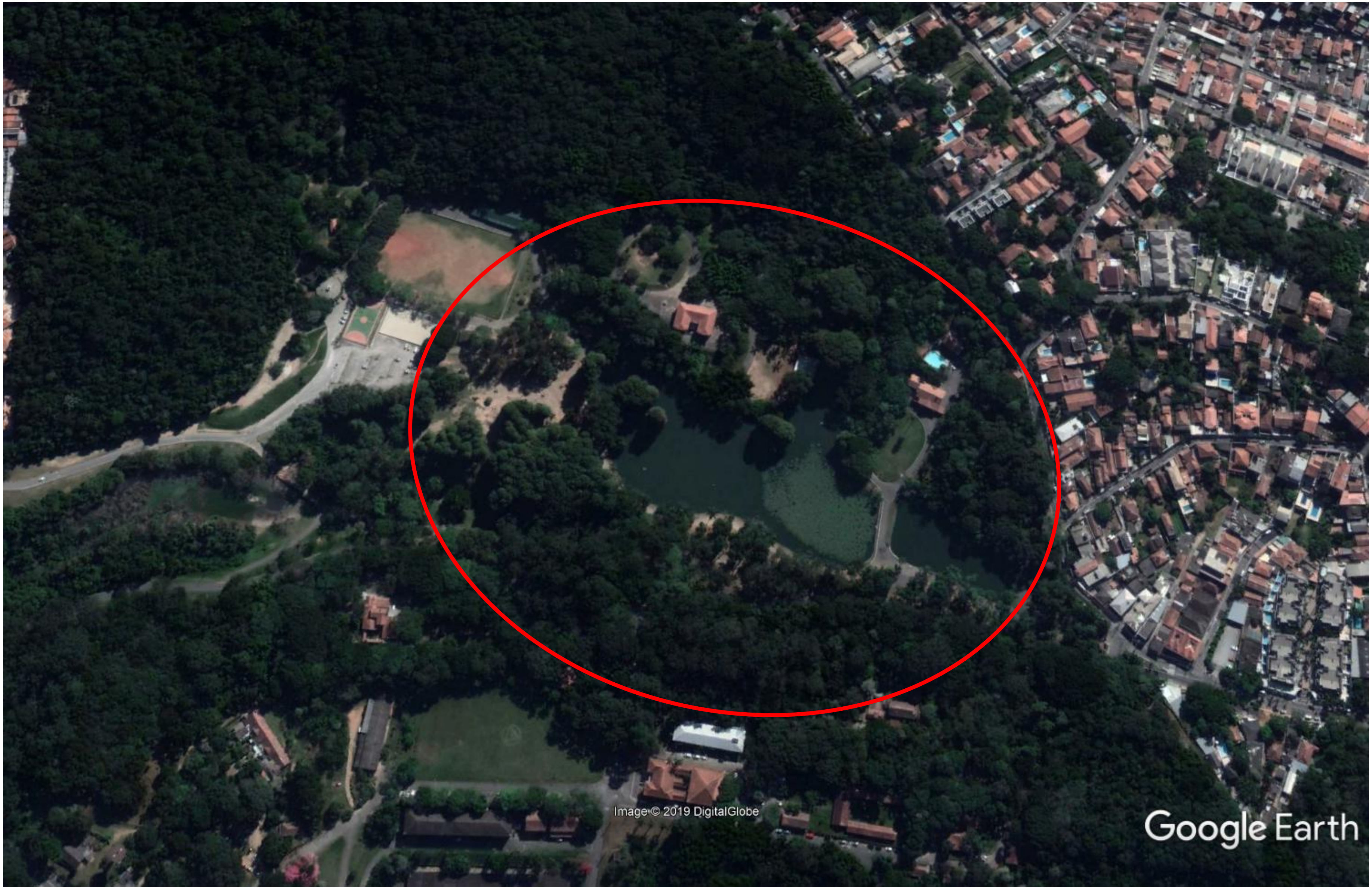

8-Poconé. Natural area (elevation of the sight point: 771 m).
Red circle indicates the area where capybaras and host-questing ticks were sampled in this study.

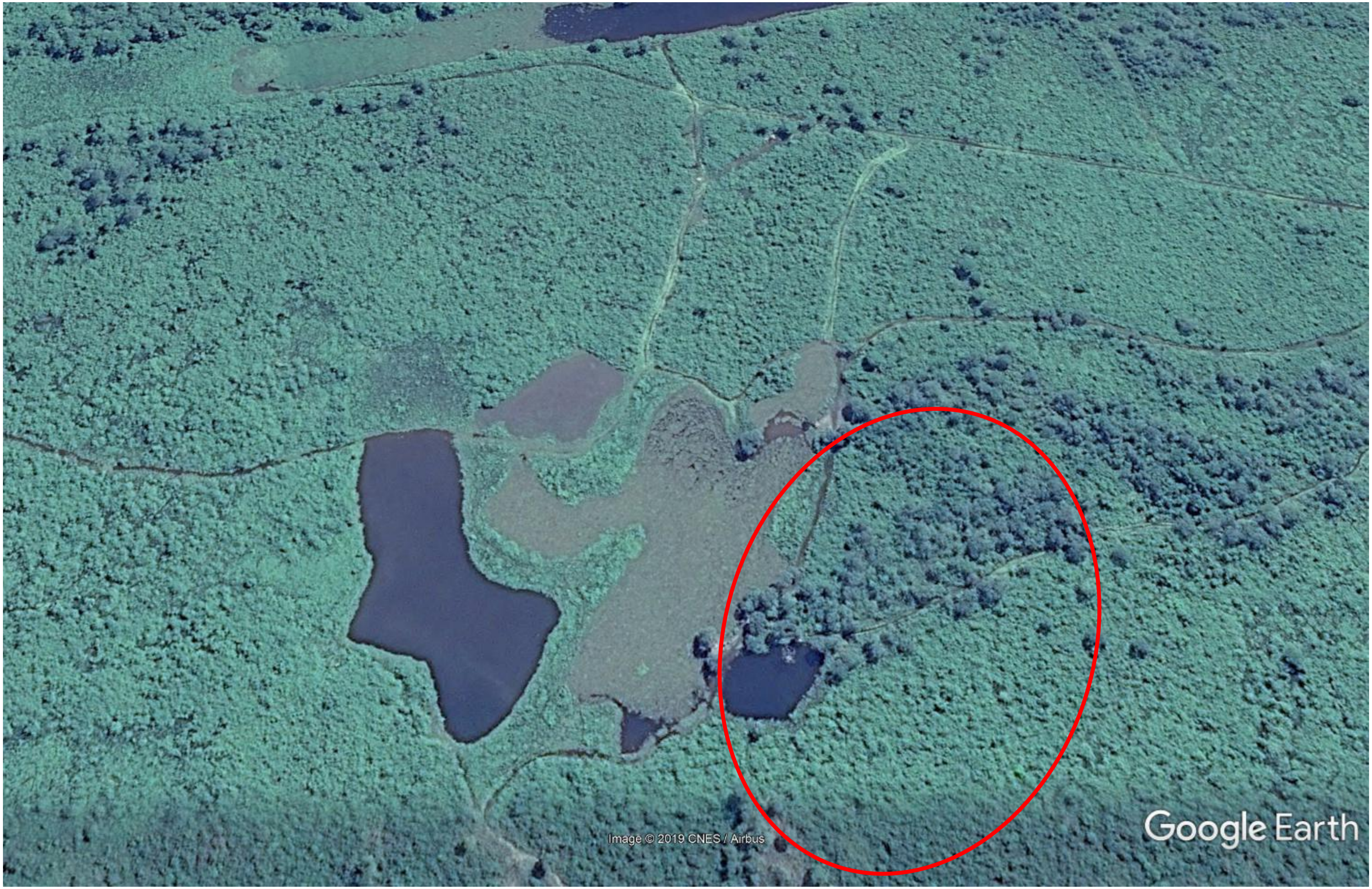

9-Corumbá. Natural area (elevation of the sight point: 1000 m).
Red circle indicates the areas where capybaras and host-questing ticks were sampled in this study.

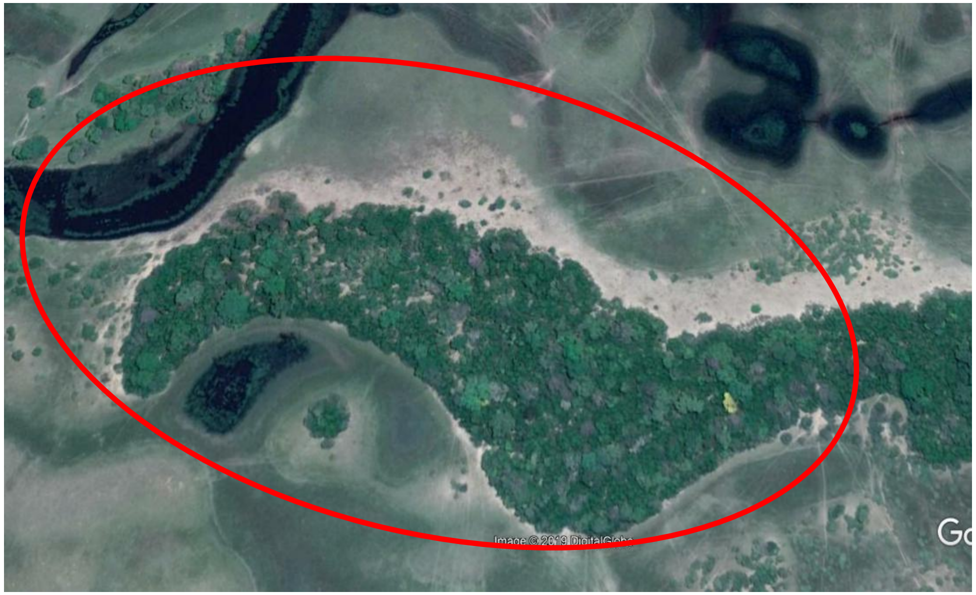

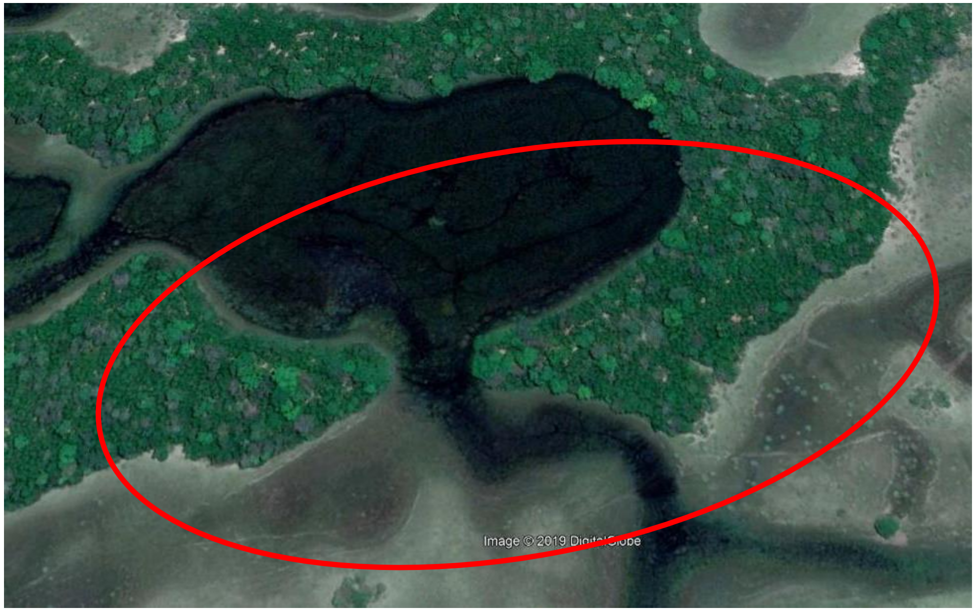

Supplement: S1 Text — Satellite images were obtained from Google Earth Pro version 7.3, and the final figure was constructed with the use of Microsoft Power Point 2010, version 14.0.7232.5000. (PDF) [file pntd.0007734.s001.pdf]
