# Supplementary material for: Epidemiology of capybara-associated Brazilian spotted fever
Source: PLoS Negl Trop Dis. 2019 Sep 6;13(9):e0007734. doi: 10.1371/journal.pntd.0007734 (PMC6750615; doi:10.1371/journal.pntd.0007734)
Supplement: S1 Table — (PDF) [file pntd.0007734.s002.pdf]

S1 Table. Areas where capybaras and ticks were sampled in the present study.

Area number - Municipality (State) [a]	Epidemiological classification [b]	Description
1-Piracicaba (São Paulo)	Endemic for BSF	Highly anthropic area in the University of São Paulo campus of Piracicaba; human-modified landscape composed by sugar cane and corn crops interposed by livestock artificial pastures and degraded riparian forests along the Piracicaba River. Elevation around 586 m.
2-Americana (São Paulo)	Endemic for BSF	Highly anthropic area in the Carioba sewage treatment station; human-modified landscape composed by abandoned grass fields (no livestock) and degraded riparian forests along the Piracicaba River. Elevation around 511 m.
3-Araras (São Paulo)	Endemic for BSF	Highly anthropic area in the Federal University of São Carlos campus of Araras; human-modified landscape composed by sugar cane crops interposed by livestock artificial pastures and regenerating riparian forests along an artificial lake. Elevation around 659 m.
4-Pirassununga-A (São Paulo)	Nonendemic for BSF	Highly anthropic area in the University of São Paulo campus of Pirassununga; human-modified landscape composed by sugar cane and corn crops interposed by livestock artificial pastures and a regenerating riparian forests along an artificial lake (Captação Lake). Elevation around 596 m.
5-Pirassununga-B (São Paulo)	Nonendemic for BSF	Highly anthropic area in the University of São Paulo campus of Pirassununga; human-modified landscape composed by corn crops interposed by livestock artificial pastures and degraded riparian forests along an artificial lake (Risca Faca Lake). Elevation around 619 m.
6-Ribeirão Preto (São Paulo)	Nonendemic for BSF	Highly anthropic area in the University of São Paulo campus of Ribeirão Preto; human-modified landscape composed by grass fields (no livestock) and degraded forests along two streams. Elevation around 550 m.
7-São Paulo (São Paulo)	Nonendemic for BSF	Highly anthropic area in the Alberto Löfgren State Park; human-modified landscape composed by grass fields (no livestock) and Atlantic forest along an artificial lake. Elevation around 900 m.
8-Poconé (Mato Grosso)	Natural area	Natural landscape composed by seasonally flooded scrublands and forests, few above high-water level forests interposed by natural lakes and rivers; no livestock or open grass lands. Elevation around 125 m.
9-Corumbá (Mato Grosso do Sul)	Natural area	Natural landscape composed by seasonally flooded grass lands (natural pastures) interposed by natural lakes and high-water level forests; livestock present at low densities. Elevation around 105 m.

[a] area numbers 1 to 9 are indicated in the map of Fig. 1
[b] classification according to official data from Brazilian Ministry of Health (http://portalsinan.saude.gov.br/dados-epidemiologicos-sinan) and local studies in each of the selected areas regarding the occurrence of Brazilian spotted fever (BSF) [Horta et al. 2007, Perez et al. 2008, Mazzei et al. 2009, Brites-Neto et al. 2013, Melo et al. 2016, Corrêa-Pacheco et al. 2017, Rocha et al. 2017]; natural areas are pristine locations of the Pantanal biome

References

Horta MC, Labruna MB, Pinter A, Linardi PM, Schumaker TT. Rickettsia infection in five areas of the state of São Paulo, Brazil. Mem Inst Oswaldo Cruz. 2007; 102: 793-801. http://dx.doi.org/10.1590/S0074-02762007000700003

Perez CA, Almeida AF, Almeida A, Carvalho VHB, Balestrin DC, Guimarães MS, et al. Carrapatos do gênero *Amblyomma* (acari: ixodidae) e suas relações com os hospedeiros em área endêmica para febre maculosa no estado de São Paulo. Rev Bras Parasitol Vet. 2008; 17: 210-217.

Mazzei K, Rosa AR, Arromba AL, Duarte AMC, Barleta C, Waldman CCS, et al. Levantamento e propostas de ação para as principais zoonoses dos parques estaduais Alberto Löfgren e da Cantareira. IF Sér. Reg., São Paulo, 2009. p. 25-41.

Brites-Neto J, Nieri-Bastos FA, Brasil J, Duarte KMR, Martins TF, Veríssimo CJ, et al. Environmental infestation and rickettsial infection in ticks in a Brazilian spotted fever-endemic area. Rev Bras Parasitol Vet. 2013; 22: 367-372. http://dx.doi.org/10.1590/S1984-29612013000300008

Melo AL, Witter R, Martins TF, Pacheco TA, Alves AS, Chitarra CS, et al. A survey of tick-borne pathogens in dogs and their ticks in the Pantanal biome, Brazil. Med Vet Entomol. 2016; 30:112-116. doi: 10.1111/mve.12139.

Corrêa Pacheco F; Moraes-Filho J; Rocha VJ, Sampieri BR, Zaniolo MM, Pachaly JR, Otutumi LK, Vidotto O, Labruna MB, Gonçalves DD. Anti-*Rickettsia rickett*sii antibodies in capybaras (*Hydrochoerus hydrocha*eris Linnaeus, 1766) from an agricultural landscape in Araras, São Paulo, Brazil. Semina: Ciências Agrárias, Londrina 2017; 38:2543-2550. doi: 10.5433/1679-0359.2017v38n4Supl1p2543.

Rocha VJ, Sekiama ML, Gonçalves DD, Sampieri BR, Barbosa GP, Dias TC, et al. Capivaras (*Hydrochoerus hydrochaeris*) e a presença do carrapato (*Amblyomma sculptum*) no campus sa UFSCAR-Araras, São Paulo. Cienc. anim. bras., Goiânia 2017; 18:e-44671, 2017. doi: 10.1590/1089-6891v18e-44671.
