# Supplementary material for: Epidemiology of capybara-associated Brazilian spotted fever
Source: PLoS Negl Trop Dis. 2019 Sep 6;13(9):e0007734. doi: 10.1371/journal.pntd.0007734 (PMC6750615; doi:10.1371/journal.pntd.0007734)
Supplement: S2 Table — (PDF) [file pntd.0007734.s003.pdf]

S2 Table. Antibody endpoint titers determined by immunofluorescence assays (IFA) against antigens of six *Rickettsia* species in sera of capybaras captured in 9 localities, being 3 Brazilian spotted fever (BSF)-endemic areas, 4 BSF-nonendemic areas, and 2 natural areas of Brazil, during 2015-2018.

Areas	Range of endpoint titers (Median in parenthesis)					
	Rickettsia rickettsii	*Rickettsia parkeri*	*Rickettsia amblyommatis*	*Rickettsia rhipicephali*	*Rickettsia bellii*	*Rickettsia felis*
BSF-endemic areas						
1-Piracicaba	128 – 16384 (512)	64 – 1024 (128)	64 – 512 (128)	64 – 512 (128)	64 – 512 (128)	64 – 512 (64)
2-Americana	64 – 2048 (256)	64 – 1024 (128)	64 -256 (128)	64 – 512 (128)	64 – 1024 (128)	64 – 256 (64)
3-Araras	64 – 2048 (128)	64 – 512 (64)	64 – 512 (128)	64 – 128 (64)	64 – 1024 (128)	64 – 128 (64)
BSF-nonendemic areas						
4-Pirassununga-A	64 – 128 (64)	64 – 256 (64)	64 – 128 (64)	64 – 128 (64)	64 -512 (128)	64 – 128 (96)
5-Pirassununga-B	64 – 128 (64)	64 – 128 (64)	64 (64)	64 (64)	64 -1024 (64)	64 (64)
6-Ribeirão Preto	64 – 128 (64)	64 – 256 (64)	64 – 256 (64)	64 – 256 (64)	64 – 512 (64)	64 (64)
7-São Paulo	64 (64)	64 (64)	64 – 128 (96)	64 (64)	64 -512 (128)	
Natural areas						
8-Poconé	64 – 512 (128)	64 – 1024 (256)	256 – 1024 (512)	64 – 512 (256)	64 – 1024 (256)	64 – 512 (64)
9-Corumbá	64 – 256 (64)	64 – 256 (64)	64 – 512 (64)	64 – 128 (64)	256 – 512 (128)	
