# Supplementary material for: Epidemiology of capybara-associated Brazilian spotted fever
Source: PLoS Negl Trop Dis. 2019 Sep 6;13(9):e0007734. doi: 10.1371/journal.pntd.0007734 (PMC6750615; doi:10.1371/journal.pntd.0007734)
Supplement: S3 Table — (PDF) [file pntd.0007734.s004.pdf]

S3 Table. Host-questing ticks collected by dry-ice traps in 9 localities [3 Brazilian spotted fever (BSF)-endemic areas, 4 BSF-nonendemic areas, and 2 natural areas] during 2015-2019.

Areas	No. Traps	*A. sculptum*					*A. dubitatum*					*A. parvum*				*O. rostratus*				Total	Total No. ticks/ trap
		Larvae	Nymphs	Adults	Total	No. ticks/ trap	Larvae	Nymphs	Adults	Total	No. ticks/ trap	Nymphs	Adults	Total	No. ticks/ trap	Nymphs	Adults	Total	No. ticks/ trap		
**BSF-endemic areas**																					
**1-Piracicaba**	**40**	2	449	836	1287	32.2		7	3	10	0.3									1297	32.4
**2-Americana**	**20**		777	586	1363	68.2	12	369	47	428	21.4									1791	89.6
**3-Araras**	**20**	2	45	133	180	9.0	10	269	35	314	15.7									494	24.7
**Total**	**80**	**4**	**1271**	**1555**	**2830**	**35.4**	**22**	**645**	**85**	**752**	**9.4**									**3582**	**44.8**
**BSF-nonendemic areas**																					
**4-Pirassununga-A**	**20**	2	650	33	685	34.3		708	8	716	35.8									1401	70.1
**5-Pirassununga-B**	**20**		1050	43	1093	54.7		1193	30	1223	61.2									2316	115.8
**6-Ribeirão Preto**	**40**		644	76	720	18.0		381	4	385	9.6									1105	27.6
**7-São Paulo**	**20**		7	10	17	0.9	6	27	21	54	2.7									71	3.6
**Total**	**100**	**2**	**2351**	**162**	**2515**	**25.2**	**6**	**2309**	**63**	**2378**	**23.8**									**4893**	**48.9**
**Natural areas**																					
**8-Poconé**	**20**		80		80	4.0		12		12	0.6									92	4.6
**9-Corumbá**	**20**		93	78	171	8.6						10	4	14	0.7	3	35	38	1.9	223	11.2
**Total**	**40**	**0**	**173**	**78**	**251**	**6.3**	**0**	**12**	**0**	**12**	**0.3**	**10**	**4**	**14**	**0.4**	**3**	**35**	**38**	**1.0**	**315**	**7.9**
**TOTAL**	**220**	**6**	**3795**	**1795**	**5596**	**25.4**	**28**	**2966**	**148**	**3142**	**14.3**	**10**	**4**	**14**	**0.1**	**3**	**35**	**38**	**0.2**	**8790**	**40.0**
